# Actin-membrane interface stress regulates Arp2/3-branched actin density during lamellipodial protrusion

**DOI:** 10.64898/2026.03.06.710140

**Published:** 2026-03-16

**Authors:** Mitchell T. Butler, Max A. Hockenberry, Harrison H. Truscott, Wesley R. Legant, James E. Bear

**Affiliations:** 1 UNC Lineberger Comprehensive Cancer Center, University of North Carolina School of Medicine, Chapel Hill, North Carolina; 2 Department of Cell Biology and Physiology, University of North Carolina School of Medicine, Chapel Hill, North Carolina; 3 Department of Pharmacology, University of North Carolina School of Medicine, Chapel Hill, North Carolina; 4 Lampe Joint Department of Biomedical Engineering, University of North Carolina at Chapel Hill and North Carolina State University, Chapel Hill, NC

## Abstract

Motile cells can sense and exert forces on the extracellular environment through dynamic actin networks. Increased stress against the polymerizing barbed ends of branched actin networks has been shown to lead to an increase in the density of these networks through a force feedback mechanism, though this phenomenon has not been explored through the examination of real-time responses of endogenous actin networks in cells. Here, we utilize mouse embryonic fibroblast CRISPR knock-in lines with labeled ARP2/3 complex to identify cellular and extracellular conditions that regulate branched actin density and enrichment at the leading edge of lamellipodial protrusions. A common theme shared among all branched actin density-increasing conditions is higher levels of interface stress between the plasma membrane and the barbed ends of the lamellipodial actin network. Among these conditions, we find that ARP2/3 is specifically required for robust spreading and protrusion in response to increased extracellular viscosity. Interestingly, time-lapse traction force microscopy of ARP2/3-dependent viscosity responses show significantly reduced changes in strain energy applied to the substrate when compared to spreading and motility through cell-matrix adhesion. In addition, we find that increased extracellular viscosity can bypass the need for extracellular matrix proteins to support lamellipodial protrusion driven by optogenetic Rac activation. Our studies provide strong support for *in vitro* models of branched actin force feedback responses and further characterize an essential role for branched actin in mediating dramatic cell shape changes in response to increased extracellular viscosity.

## INTRODUCTION

The ability of cells to sense and respond to their environment is essential for many physiological processes such as wound healing(Shaw and Martin 2016), immune response(Friedl and Weigelin 2008, Ryan, Kim et al. 2024), neuronal pathfinding(Ayala, Shu et al. 2007), and tissue morphogenesis(Aman and Piotrowski 2010). Cells can interact with their extracellular environment through transmembrane receptors, and among these, cell adhesion receptors such as integrins connect cytoskeletal networks through cytoplasmic adaptor proteins to the extracellular matrix (ECM) to govern cell shape and behavior(Parsons, Horwitz et al. 2010, Kechagia, Ivaska et al. 2019). The linkage of ECM to the actin cytoskeleton through integrin-containing adhesions serves as a molecular clutch and allows motile cells to push and pull against the substrate, producing the traction forces that facilitate cellular translocation(Lauffenburger and Wells 2001). Importantly, cells adapt the distribution and magnitude of forces they apply on the substrate based on extracellular molecular signals and the physical properties of the environment, though there is still much to uncover regarding the hierarchy, feedback, and overlap between various mechanistic inputs.

While most studies on links between integrins and actin regulation have primarily focused on contractile actomyosin bundles anchored to larger, mature focal adhesions, smaller, more dynamic nascent adhesions associated with polymerizing branched actin networks also play an important role in sensing environmental cues to direct cell migration(Romero, Le Clainche et al. 2020). Highly branched dendritic actin networks can be found in lamellipodia, where their polymerization against the membrane provides the force to not only push the leading cell edge forwards(Pollard and Borisy 2003), but also to cluster small groups of integrin receptors near the edge(Choi, Vicente-Manzanares et al. 2008). The seven-subunit Arp2/3 complex can bind to the side of an existing actin filament and nucleate a new filament as a stereotyped branch with an angle of ~70°(Mullins, Stafford et al. 1997, Svitkina and Borisy 1999). Dense branched actin networks are essential for cells to sense and respond to gradients of ECM proteins(Wu, Asokan et al. 2012), and during these haptotactic responses, cell adhesion signaling promotes more persistent protrusions and migration towards higher ECM concentrations through the activity of FAK and Src family kinases(King, Asokan et al. 2016). Downstream signaling leads to activation of Rac GTPase, which activates the WAVE regulatory complex (WRC) to promote the formation Arp2/3-actin branches at the leading edge of protrusions(Rotty, Wu et al. 2013, Lappalainen, Kotila et al. 2022).

In addition to molecular signaling, there is increasing evidence that physical forces can also impact Arp2/3-branched actin dynamics(Romero, Le Clainche et al. 2020, Lappalainen, Kotila et al. 2022). Several *in vitro* studies have highlighted a force feedback mechanism that increases the density of branched actin networks polymerized against increasing compressive forces(Bieling, Li et al. 2016, Bieling, Weichsel et al. 2022). In cells, aspiration or severing the rear of cells to manipulate membrane tension can positively or negatively influence leading edge actin density, respectively(Mueller, Szep et al. 2017), and qualitative observations of similarly increased leading edge actin density have been made when artificially increasing membrane tension(Gauthier, Fardin et al. 2011) or extracellular viscosity(Bera, Kiepas et al. 2022). Branched actin is also essential for cell volume control in response to osmotic stresses(Wu, Haynes et al. 2013), likely mediated through changes in actin cortex when global forces are applied to cells. However, our understanding of how forces influence branched actin has been limited by the tools available to directly observe branch formation in cells and quantitative comparisons of various mechanical inputs.

While these studies point to roles for either signaling through integrin adhesions or physical forces on Arp2/3-branched actin regulation, few studies have explored the overlap or interplay between load force and adhesion signaling, and contributions that are shared or unique between them. We previously developed tools allowing for the optogenetic activation of Rac(Guntas, Hallett et al. 2015) and employed them in cells on a wide range of substrate plating conditions(Zimmerman, Asokan et al. 2017). From this, we found that a minimum threshold of ECM concentration must be met in order for cells to protrude in response to optogenetic Rac activation. However, we were left with several open questions, such as whether protrusion failed on low ECM conditions due to insufficient signaling, failed mechanical clutching of actin networks through integrins adhesions, or some combination of both.

By using cells with endogenously tagged Arp2/3 complex, we addressed these questions here with live-cell analyses of actin branch formation under conditions that increase the “load force” on barbed ends of the dendritic lamellipodial actin network pushing against the plasma membrane. From these experiments, we offer a detailed and quantitative characterization of Arp2/3-branched actin dynamics in response to changes at the actin-membrane interface and its relationship to signaling downstream of nascent integrin adhesions. Cells that adopt spread, flattened shapes upon ECM engagement, relaxation of cortical actomyosin, or application of external physical forces all exhibit increasingly enriched branched actin at the edge of flat protrusions, and additionally, cells subjected to osmotic shock or extracellular viscosity show similar effects. Genetic loss of Arp2/3 showed that branched actin specifically is required for dramatic cell shape changes in response to increased extracellular viscosity, and in turn, increased extracellular viscosity can support both efficient cell spreading and successful protrusion following optogenetic Rac activation on low ECM conditions, shedding further light the close relationship between branched actin and mechanical forces acting on cells.

## RESULTS

### Cells with endogenously labeled Arp2/3 complex reveal striking substrate-dependent actin network architecture at the leading edge

To assess how branched actin dynamics are influenced by extracellular matrix (ECM) to control cell shape and behavior, we employed a mouse dermal fibroblast cell line with the Arpc2 (p34) subunit of the Arp2/3 complex endogenously labeled with a mScarlet red fluorescent protein via CRISPR/Cas9 gene editing. Biallelic labeling of this subunit allows for live-cell visualization of all endogenous Arp2/3 complexes. To evaluate the impact of ECM engagement on Arp2/3 spatiotemporal dynamics, these fibroblasts were seeded on glass coverslips coated with either dense fibronectin ECM to engage integrins or poly-L-Lysine (PLL), which promotes cell flattening and spreading through electrostatic change differences between the lysine residues and the cell surface. It is important to note that these cells also cannot produce their own fibronectin due to a CRISPR-induced disruption of the *Fn1* gene (Chandra, Butler et al. 2022).

**Figure 1.**
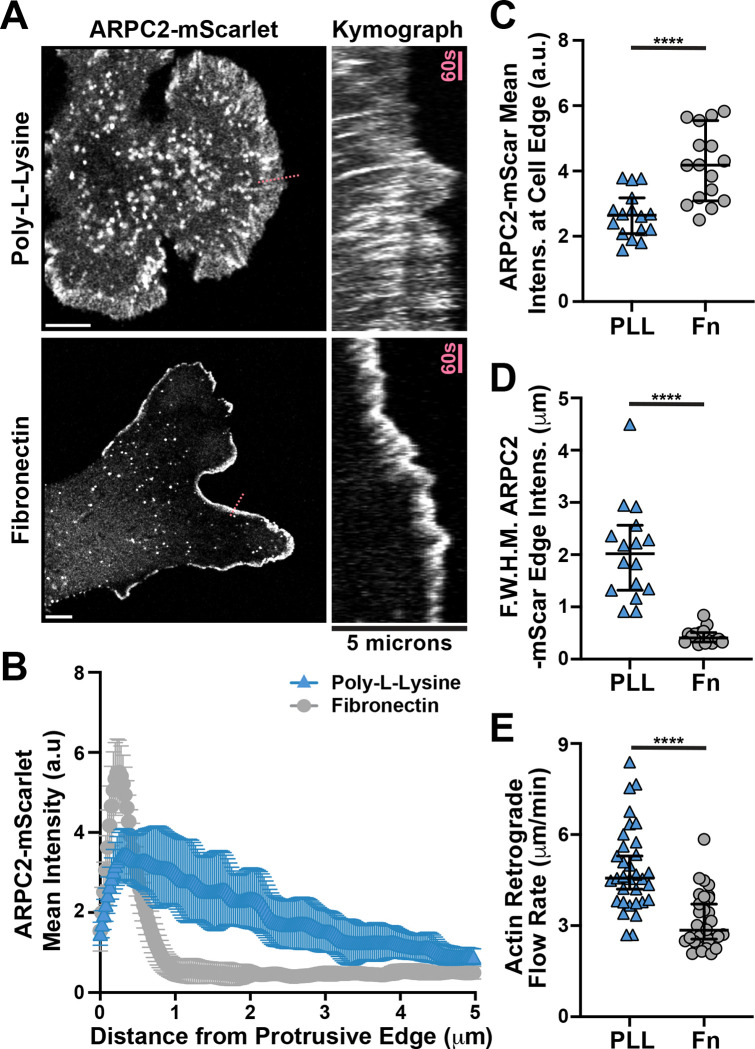
Cells with endogenously labeled Arp2/3 complex reveal striking substrate-dependent actin network architecture at the leading edge. **(A)** Frames from time lapse movies of endogenously-labeled ARPC2-mScarlet expressed by Fibroblasts plated on glass coverslips coated with either fibronectin or poly-L-Lysine alongside associated kymographs of protrusive regions marked with a dashed pink line in the left panels. Scale bars span 10 microns. **(B)** Plot of mean ARPC2-mScarlet intensities along line scans starting near the middle of the largest cellular protrusion, aligned perpendicular to the cell edge, and directed inwards for 5 microns while avoiding regions of obvious ruffles and folds in cells plated as described in (A). n = 16 cells from 3 experiments **(C)** Plot of mean ARPC2-mScarlet intensities at and within roughly 1 micron of the cell edge along the largest protrusion in fibroblasts plated as described in (A). n = 16 cells from 3 experiments **(D)** Plot of width measurements in microns of ARPC2-mScarlet signal that is above 50% the maximum intensity recorded, referred to as “Full Width Half Max” and abbreviated as F.W.H.M, in the line scans shown and described in (B). **(E)** Plot of retrograde flow rates visualized by bleaching GFP-β-Actin signal at the edge of protrusions in cells plated as described in (A) and measured by calculating the slope of recovering signal in kymographs generated from bleached regions. n = 33 and 32 cells for PLL and Fn conditions, respectively, from 3 experiments. Note that the data from cells on poly-L-Lysine coated glass in panels (B-E) were collected at the same time as other experimental conditions for a separate study, and values for fibronectin here have been published as the 10 ug/ml control condition previously (Chandra, Butler et al. 2022).

Roughly one hour after plating, cells on poly-L-Lysine exhibited a broad, irregular distribution of Arp2/3, appearing as dense accumulations surrounded by more diffuse regions along the leading edge of flat protrusions (Fig.1A and Movie 1). A striking difference was seen in cells plated instead on fibronectin-coated substrates, which showed a dense and uniform enrichment of ARPC2-mScarlet at the leading edge of lamellipodia, labeling the highly branched dendritic actin network observed in fibroblasts (Svitkina and Borisy 1999) (Fig.1A and Movie 2). Consistent with our visual impressions, line scans of the cell periphery show a sharper, taller peak when cells are plated on fibronectin compared to a wider distribution with a more gradual drop-off in intensity when plated on poly-L-Lysine (Fig.1B). Drawing a line ~1 micron wide along the furthest edge of the largest cell protrusions and measuring the mean intensity in this region serves as a general measure of Arp2/3 enrichment at the leading edge, which is nearly double the value for cells plated on poly-L-Lysine when compared to cells plated on fibronectin (Fig.1C). From ARPC2-mScarlet line scans (Fig.1B), we also calculated the width of the region with a mean intensity over half the maximum peak value measured, referred to as the full width half max value (FWHM). This Arp2/3 width difference between the two plating conditions supports the notion that actin branching density is significantly higher at the leading edge of cells plated on fibronectin, relative to PLL (Fig.1D). As expected with an increase in Arp2/3 density at the leading edge, the amount of free barbed ends is significantly increased in cells plated on fibronectin compared to poly-L-Lysine (Fig.1S1A-C), confirming that the more abundant Arp2/3 complexes seen here are being incorporated into the leading edge actin network and mediating more dense actin branching.

### Branched actin enrichment in fibroblasts plated on fibronectin depends on Integrin engagement.

We previously demonstrated that clusters of nascent or immature adhesions labeled by GFP-Paxillin are reduced in number when fibroblasts plated on lower fibronectin concentrations(Chandra, Butler et al. 2022), and we were able to verify that such structures are essentially absent in protrusions over poly-L-Lysine (Fig.1S3A-B). Because a reduction of Paxillin-positive adhesive structures would presumably reduce the extent of actin retrograde flow clutching, it is likely that the more diffuse and broader distribution of Arp2/3 from the leading edge is concomitant with an increase in retrograde flow rates. Indeed, upon bleaching GFP-β-actin at the leading edge and monitoring the dynamic retrograde flow of subsequently polymerized F-actin, we measured significantly faster flow rates in cells plated on poly-L-Lysine relative to the dense Fn plating condition (Fig.1E).

To confirm the role of integrin adhesions specifically on leading edge actin organization and examine the potential influence of alternate or secondary signaling pathways through proteins such as Syndecans(Couchman and Woods 1999, Midwood, Valenick et al. 2004), we utilized coverslips coated with purified RGD peptides. Cells plated on glass coated with either fibronectin or RGD peptides had abundant focal adhesions and actin stress fibers, both of which were lacking in cells plated on poly-L-Lysine (Fig.1S3C). Measuring the mean Arpc2-mScarlet intensity along the very leading edge revealed that compared to cells plated on poly-L-Lysine, there was an increase in Arp2/3 enrichment on RGD-coated glass that was comparable to the increase in enrichment seen on fibronectin (Fig.1S3C-D). Thus, the stark differences seen in branched actin organization in cells plated on fibronectin when compared to poly-L-Lysine is likely mediated through engagement with integrin-based adhesions.

### The requirement for the Arp2/3 complex in poly-L-Lysine-based protrusions

**Figure 2.**
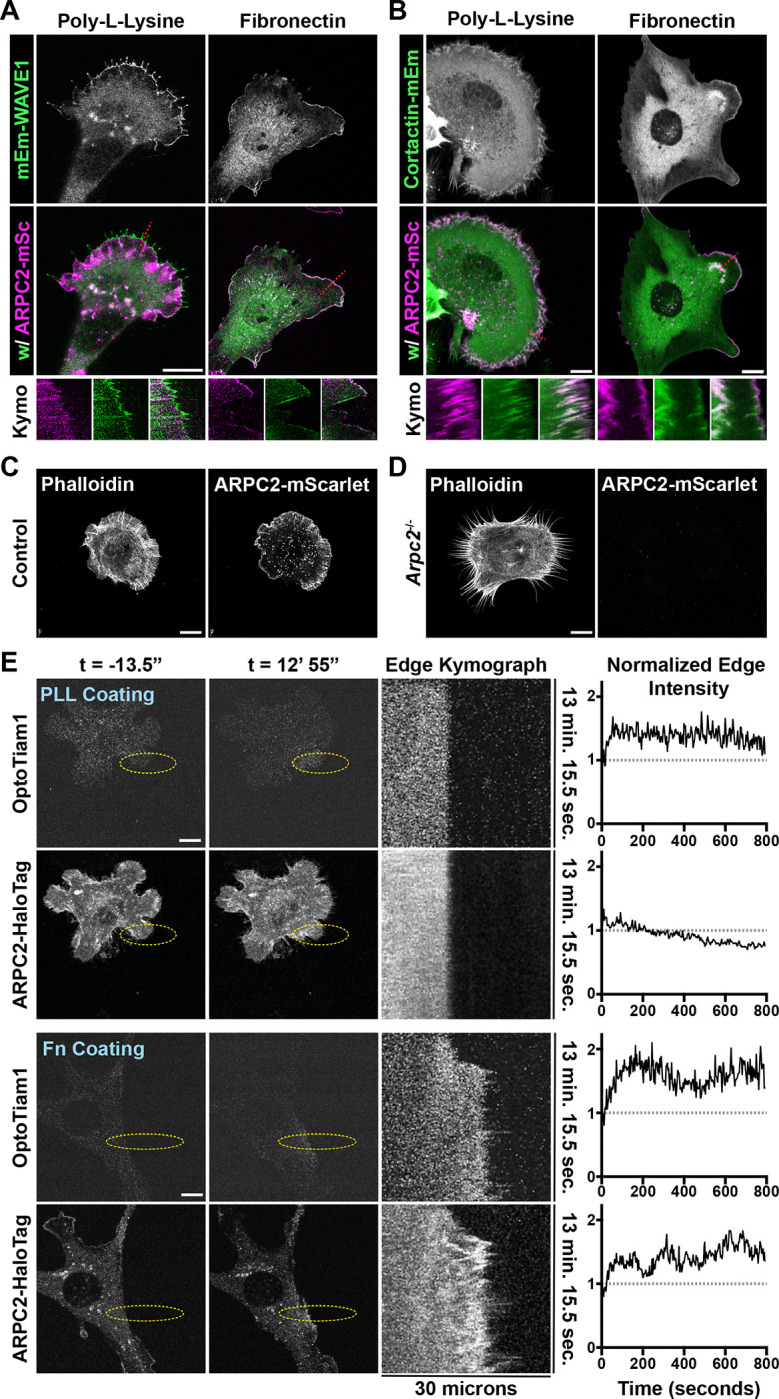
Increased Rac activation is not sufficient to induce dense branched actin organization on PLL. **(A)** Frames from time lapse movies of endogenously-labeled ARPC2-mScarlet and lentiviral-transduced mEmerald-WAVE1 expressed by Fibroblasts plated on glass coverslips coated with either fibronectin or poly-L-Lysine and associated kymographs of protrusive regions marked with a dashed red line. Scale bars span 10 microns. **(B)** Frames from time lapse movies of endogenously-labeled ARPC2-mScarlet and lentiviral-transduced Cortactin-mEmerald expressed by Fibroblasts plated on glass coverslips coated with either fibronectin or poly-L-Lysine and associated kymographs of protrusive regions marked with a dashed red line. Scale bars span 10 microns. **(C)** Images of control fibroblasts with Arp2/3 intact plated on poly-L-Lysine coated surfaces and visualized using fluorescently-labelled phalloidin. Scale bar spans 10 microns. **(D)** Images of 4HT-treated *Arpc2* knockout fibroblasts plated on poly-L-Lysine coated surfaces and visualized using fluorescently-labelled phalloidin. Scale bar spans 10 microns. **(E)** Images from timelapse movies of Tiam1-DH/PH-TagRFPt-SspBmicro and ARPC2-HaloTag (labeled with JF646 Halo Ligand) stably expressed by 4HT-treated *Arpc2* knockout fibroblasts via lentiviral transduction that have been plated on a glass coverslip coated with poly-L-Lysine (top panels) or fibronectin (bottom panels) and regularly stimulated with 405 nm light inside the region of interest (ROI) labeled with a yellow dashed oval starting at t = 0. These cells additionally express Venus-iLid-caax (not shown). The associated kymograph was generated from a line (not shown) bisecting the yellow dashed oval ROI along the long axis, and the associated plot is a measure of the intensity of the associated marker within ~1 micron of the cell edge down the length of the accompanying kymograph. Scale bars span 10 microns.

The lack of dense Arp2/3-branched actin observed on PLL could be due to reduced Arp2/3-branch generation via nucleation promoting factors (NPF) or insufficient branch stabilization. To test these possibilities, we examined the localization of WAVE1 (NPF) and Cortactin (branch stabilizer) when cells were plated on PLL. Regardless of whether cells were plated on poly-L-Lysine or fibronectin, we observed abundant WAVE1 at the cell periphery (Fig.2A and Movies 3-4) and Cortactin (Fig.2B) throughout the actin network during protrusion, exhibiting similar localization and dynamics to labeled Arp2/3, suggesting that differences in these factors is unlikely to explain the observed differences in branched actin organization. We also tested the functional requirement for Arp2/3 in producing the broad lamellipodia-like protrusions observed on PLL. To accomplish this, we induced Cre-mediated recombination at the conditional *Arpc2* locus and plated cells on PLL. Control cells (Arp2/3 intact) plated on poly-L-Lysine displayed the expected wide, cohesive protrusions with sparse Arp2/3 branching (Fig.2C), but Arp2/3 null cells displayed only narrow bundled actin protrusions, appearing as separate structures lacking the more diffusively labeled meshwork between the bundled actin structures (Fig.2D). We conclude that differences seen in branched actin density between PLL and fibronectin are not due to an inability for branches to be nucleated or stabilized on PLL, since broad Arp2/3-dependent protrusions are still present, but instead may rely on differences in either signaling magnitude and/or force feedback on the branched actin network.

### Increased Rac activation is not sufficient to induce dense branched actin organization on PLL.

Although cells plated on PLL have Arp2/3-activating NPFs such as WAVE at the leading edge, one possible explanation for the sparse density of branched actin observed under these conditions is insufficient integrin-mediated signaling that activates the NPFs at the leading edge. Signaling from nascent integrin adhesions at the leading edge activates kinases such as FAK and Src, leading to Rac activation(Choi, Zareno et al. 2011, King, Asokan et al. 2016). Nascent adhesions were markedly reduced or absent in protrusions over poly-L-Lysine (Fig.1S2). We previously demonstrated that activation of endogenous Rac by recruiting the DHPH domain of the RacGEF Tiam1 via iLid-based optogenetic components could not drive lamellipodial protrusions in cells plated on poly-L-Lysine despite being very effective at doing so in cell plated on fibronectin-coated surfaces (Zimmerman, Asokan et al. 2017).

To test whether Rac activation was sufficient to restore dense Arp2/3-branched actin in cells plated on PLL, we used optogenetic Rac activation in cells plated on poly-L-Lysine and assessed the enrichment of Arp2/3(Zimmerman, Asokan et al. 2017). Consistent with previous work, we found that optogenetic Rac activation does not drive robust cell protrusion on poly-L-Lysine, and here we observed negligible changes in labeled Arp2/3 distribution in stimulated regions (Fig.2E and Movie 5). In contrast, control cells extended robust cell protrusions over fibronectin driven by optogenetic Rac activation and exhibited dense branched actin within such protrusions (Fig2E and Movie 6). These results indicate that increased Rac activity is insufficient for dense actin branching in cell protrusions, and since activation of Rac has been identified as one of the key signaling events downstream of integrin signaling to support cell protrusion, this result also suggests that differences in integrin-mediated signaling alone are unlikely to account for the differences in Arp2/3 distribution in cells plated on the different substrates.

### Enrichment of Arp2/3-branched actin is linked to cell spreading

**Figure 3.**
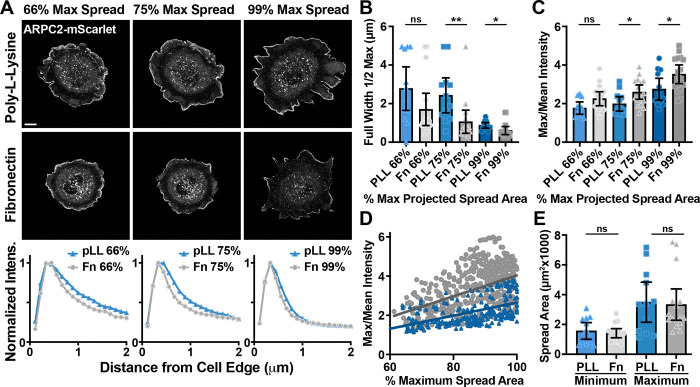
Enrichment of Arp2/3-branched actin is linked to cell spreading. **(A)** Representative images of ARPC2-mScarlet endogenously expressed by fibroblasts plated on glass coverslips coated with either poly-L-Lysine or fibronectin as they reached a projected cell spread area of 66%, 75%, and 99% of the maximum spread area recorded. Graphs below the image panels show the distribution of intensity around the cell periphery at a given distance from the cell edge for each of the two plating conditions shown above. Scale bar spans 10 microns. **(B)** Plot of Full Width Half Max ARPC2-mScarlet mean intensity around the cell periphery at various stages of spreading relative to the maximum recorded when plated on surfaces coated with poly-L-Lysine or fibronectin. **(C)** Plot of the maximum value from a range of mean Arpc2-mScarlet intensities measured in 1-pixel intervals around the cell spanning from the cell edge to 5 microns inward, which is divided by the mean value of all intensities throughout this same range,, shown as an alternative measure for the values shown in (B) and graphically detailed in Figure 3 Supplement 1. **(D)** Plot of the maximum/mean ARPC2-mScarlet intensity within 5 microns of the cell edge around the entirety of the cell periphery against cell spread area as a percentage of the maximum recorded, showing a significantly steeper slope for these values as a group when measured in cells plated on fibronectin compared to those plated on poly-L-Lysine. **(E)** Minimum and maximum projected spread areas of the cells measured. n = 12 for PLL and 16 for Fn from 3 experiments for (B-E).

To better understand the differences between substrate plating conditions, we also imaged the cells by SEM. Cells plated on FN showed completely flat, smooth protrusions while the cells on PLL showed abundant ruffles, filopodia, and folds in the plasma membrane (Fig.3S1A-B). These structures could act as “reservoirs” for excess plasma membrane in these cells and may indicate lower plasma membrane tension in cells plated on poly-L-Lysine. Furthermore, it also suggests that cells may be spreading at very different rates under these conditions, and measuring spread area over time confirmed this notion (Fig.3S1C). In extended time-lapse movies of cells plated on PLL, we observed enrichment of the Arp2/3-branched actin when the cells reached what appeared to be their maximum spread area (Fig.3S1D). Interestingly, some cells plated on PLL would spread out transiently and then shrink back to a smaller footprint, and the Arp2/3-branched actin density would enrich only during the period of maximum spreading (Fig.3S1D and Movie 7).

To quantitatively test the idea that Arp2/3-branched actin density was linked to spread area, we examined branched actin content relative to cell spread area over time in cells plated on either fibronectin or PLL (Movie 8). For this characterization, we used live-cell imaging to record the entire spreading process and generated ARPC2-mScarlet intensity profiles around the cell periphery at each time point in cells plated on either fibronectin or poly-L-Lysine. We related these values of cell spread area to the maximum area observed (Fig.3A & Fig.3S2). From these profiles, we calculated FWHM values that show cells plated on fibronectin tend to have more enriched branched actin throughout the spreading process relative to poly-L-Lysine plating, but cells plated on either substrate have branched actin that is increasingly confined to the outermost edge of cell protrusions as cells continue to spread (Fig.3B). The ratio of the maximum intensity over the mean intensity at each measured time point is also a useful metric for Arp2/3 density at the edge of cells (Fig.3S2), and these values similarly show that branched actin becomes increasingly enriched at the cell periphery throughout spreading and that cells plated on fibronectin produce denser branched actin networks sooner, possibility due to more robust clutching of retrograde actin network flow (Fig.3C-D). Importantly, we recorded very similar minimum and maximum projected cell spread areas on both substrates (Fig.3E), measuring Arp2/3 distribution while having captured cells adopting similar, fully spread states on both substates. These results suggest that membrane tension likely plays an important role in providing force feedback on the branched actin network, resisting further spreading once cells fully flatten, and that this seemingly occurs independently of the presence or absence of dense ECM coating on the substrate.

### Inducing spreading through Myosin inhibition promotes enrichment of branched actin in cell protrusions.

**Figure 4.**
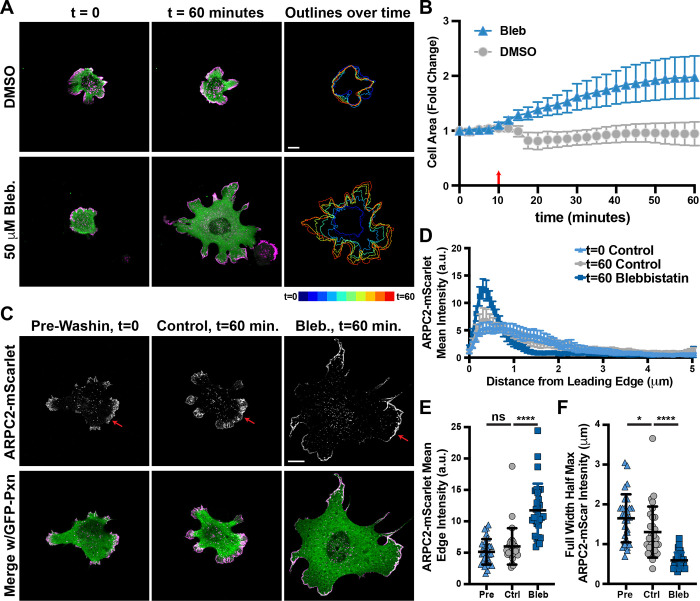
Inducing cell spreading on poly-L-Lysine with myosin inhibition results in enrichment of branched actin in cell protrusions. **(A)** Frames from time lapse movies of endogenously-labeled ARPC2-mScarlet and lentiviral transduced GFP-Paxillin expressed by Fibroblasts plated on glass coverslips coated with poly-L-Lysine and treated with either 50 μM para-amino-Blebbistatin (Bleb.) or DMSO control at t = 10 minutes showing the change in projected cell spread area over time. Scale bar spans 10 microns **(B)** Plot of projected cell spread area over time measured among cells shown and described in (A), with the red arrow at t = 10 minutes marking when wash-ins were performed. n = 25 from 2 experiments for Bleb treatment and 14 for DMSO-treated cells. **(C)** Images of cells similarly treated as described in (A) and fixed either 10 minutes prior to wash-ins or 50 minutes after for accurate measurements of ARPC2-mScarlet distribution. Scale bar spans 10 microns **(D)** Line scans of ARPC2-mScarlet intensity along lines 5 microns long and running inward from the cell edge at the largest protrusion under the indicated conditions. n = 30 cells from 2 experiments for all three groups. **(E)** Plot of mean ARPC2-mScarlet intensities at and within roughly 1 micron of the cell edge along the largest protrusion from the same cells measured for (D). **(F)** Plot of Full Width Half Max values calculated from the line scans shown in (D).

Slow spreading of cells plated on poly-L-Lysine leads to a gradual enrichment of branched actin at the cell periphery (Fig.3), and we next wanted to assess the effects of inducing rapid cell spreading on poly-L-Lysine with a similar timescale as adhesion-based spreading on surfaces coated with dense fibronectin ECM. Addition of Blebbistatin (bleb) inhibits non-muscle myosin II and should promote cells to more readily maximize their surface contact area with the electrostatically attractive poly-L-Lysine surface coating when flattening is no longer resisted by the cortical actomyosin contractility(Cai, Biais et al. 2006). Indeed, treatment of cells plated on poly-L-Lysine with 50 μM bleb led to a rapid increase in spread cell area, with around a 2-fold increase on average relative to DMSO-treated controls (Fig.4A-B and Movies 9-10).

To measure the effects of more rapid cell spreading on poly-L-Lysine on branched actin organization, bleb treatments of cells plated on poly-L-Lysine were repeated while only capturing images at the first and final time points to mitigate the effects of photobleaching (Fig.4C). The distribution of Arp2/3 in protrusions at initial one hour post-plating timepoints and in DMSO-treated controls one hour later appeared very similar to as what was seen in cells plated on poly-L-Lysine for one hour previously (Fig.1B), with shorter, shallow peaks in lines scans (Fig.4D), relatively lower intensities along the leading edge (Fig.4E), and broader distribution of signal back from the leading edge (Fig.3F). In contrast, cells plated on poly-L-Lysine and treated with bleb showed branched actin distributions very similar to cells plated on fibronectin (Fig.1B) one hour later, with tall, narrow peaks in line scans (Fig.4D), higher mean intensities along the edge of protrusions (Fig.4E), and much more narrow distribution of ARPC2-mScarlet signal confined very close to the leading edge (Fig.4F). These results suggest that forcing cells to adopt a fully spread and flattened shape is sufficient to promote dense Arp2/3-branched actin at the leading edge of protrusions.

### Physically flattening cells or manipulating membrane tension through osmotic pressure leads to enriched protrusive branched actin.

Because relieving contractility with bleb treatment can have secondary effects, such as potentially influencing F-actin turnover(Medeiros, Burnette et al. 2006, Yamashiro, Tanaka et al. 2018), we next attempted to force cells to adopt a similar spread, flattened shape while leaving non-muscle myosin II activity intact. Physically flattening cells with weighted agarose pucks on poly-L-Lysine forced cells to spread in a rapid and robust manner (Fig.4S1A-B and Movie 11). Upon physical compression, line scans demonstrate an obvious shift in the distribution of Arp2/3 to be more enriched towards the leading edge (Fig.4S1C). These finding provide additional support that cells adopting a spread, flattened shape can strongly influence branched actin density in cell protrusions.

As both bleb treatment and physical compression result in not only increased plasma membrane tension as cells spread out, but also flatter, more spatially confined cellular protrusions, we next wanted to determine whether we could potentially differentiate between the effects these two different variables, membrane tension and protrusion confinement, have on branched actin organization. Manipulation of cell volume through osmotic pressure could potentially cause less substantial cell spreading and flattening of protrusions while still changing the magnitude of the force feedback on polymerizing branched actin networks. When cells are plated on poly-L-Lysine, addition of 0.25M sorbitol to the medium introduces hyperosmotic conditions and initially caused a reduction in cell volume and spread cell area, but cells were then able to adapt and return to their original spread area roughly 30 min later and continue to spread slightly beyond their original projected area (Fig.4S1D-E and Movie 12). Despite the reduction in rate and magnitude of spreading seen, changes in the distribution of branched actin at the leading edge was very similar to both bleb and compressive treatments, with line scans and measurements of Arp2/3 density and enrichment closely resembling those seen when plating cells on fibronectin-coated substrates (Fig.4S1F-H). Interestingly, while SEM imaging reveals smooth, flattened cellular surfaces following one hour of bleb treatment, cells treated with 0.25M sorbitol typically retained abundant filopodial and ruffle-like structures seen in cells plated on poly-L-Lysine without treatment (Fig.4S1I-J). Although osmotic shock can induce a variety of cell signaling and behavior responses(Hoffmann, Lambert et al. 2009, Guo, Pegoraro et al. 2017), this serves as an additional example of experimental manipulations that increases resistance to cell protrusion and leads to actin networks with more dense branching.

### Increased extracellular viscosity can induce cell spreading in the absence of dense ECM and promotes enriched actin network branching in lamellipodial protrusions.

**Figure 5.**
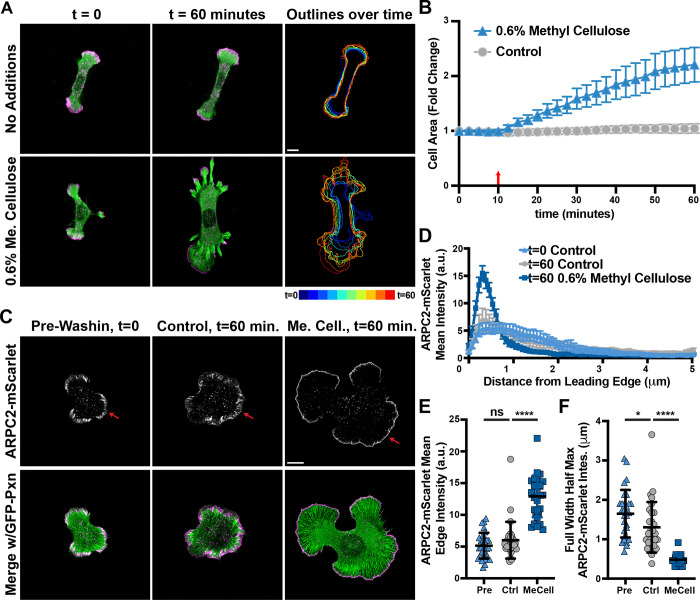
Increased extracellular viscosity can induce cell spreading in the absence of dense ECM and promotes enriched actin network branching in lamellipodial protrusions. **(A)** Frames from time lapse movies of endogenously-labeled ARPC2-mScarlet and lentiviral transduced GFP-Paxillin expressed by Fibroblasts plated on glass coverslips coated with poly-L-Lysine and either treated with 0.6% methylcellulose at t = 10 minutes or not, showing the change in projected cell spread area over time. Scale bar spans 10 microns **(B)** Plot of projected cell spread area over time measured among cells shown and described in (A), with the red arrow at t = 10 minutes marking when wash-ins were performed. n = 44 from 2 experiments for each condition. **(C)** Images of cells similarly treated as described in (A) and fixed either 10 minutes prior to wash-ins or 50 minutes after for accurate measurements of ARPC2-mScarlet distribution. Scale bar spans 10 microns **(D)** Line scans of ARPC2-mScarlet intensity along lines 5 microns long and running inward from the cell edge at the largest protrusion under the indicated conditions. n = 30 cells from 2 experiments for all three groups. **(E)** Plot of mean ARPC2-mScarlet intensities at and within roughly 1 micron of the cell edge along the largest protrusion from the same cells measured for (D). **(F)** Plot of Full Width Half Max values calculated from the line scans shown in (D).

Relieving cortical contractility, physically flattening cells, and manipulations of volume and membrane tension all similarly lead to dramatic increases in branched actin density in cell protrusions, but they all impact the cell in ways that could have potential secondary effects. To supplement these finding with an additional approach, we increased resistance to protrusion by increasing the viscosity of the media, which has been shown to promote increased cell motility and mechanical loading on peripheral branched actin networks(Bera, Kiepas et al. 2022) and promote cell spreading through a mechanism that seemingly utilizes lamellipodial ruffling(Pittman, Iu et al. 2022). The addition of methylcellulose to the media at a final concentration of 0.6% elicited a very dramatic spreading response in cells plated on poly-L-Lysine, similar in both magnitude and rate when compared to bleb treatment despite what is likely a very different mechanism of spreading induction (Fig.5A-B and Movies 13-14).

Images captured ten minutes before and fifty minutes after methylcellulose addition to the media allowed for accurate measurement of the effects of extracellular viscosity on the organization of the branched actin network (Fig.5C). The distribution of endogenously labeled Arp2/3 in cells when plated on poly-L-Lysine and induced to spread with the addition of methylcellulose showed the expected tall, narrow peaks in line scans (Fig.5D), higher mean intensities along the edge of protrusions (Fig.4E), and much more narrow distribution of ARPC2-mScarlet signal confined very close to the leading edge (Fig.5F) in comparison to untreated controls. Together, these data show that viscosity changes can induce cell spreading on PLL surfaces and consequently increase the density of the Arp2/3-branched actin network at the leading edge.

### Robust spreading in response to increased extracellular viscosity requires Arp2/3-branched actin.

**Figure 6.**
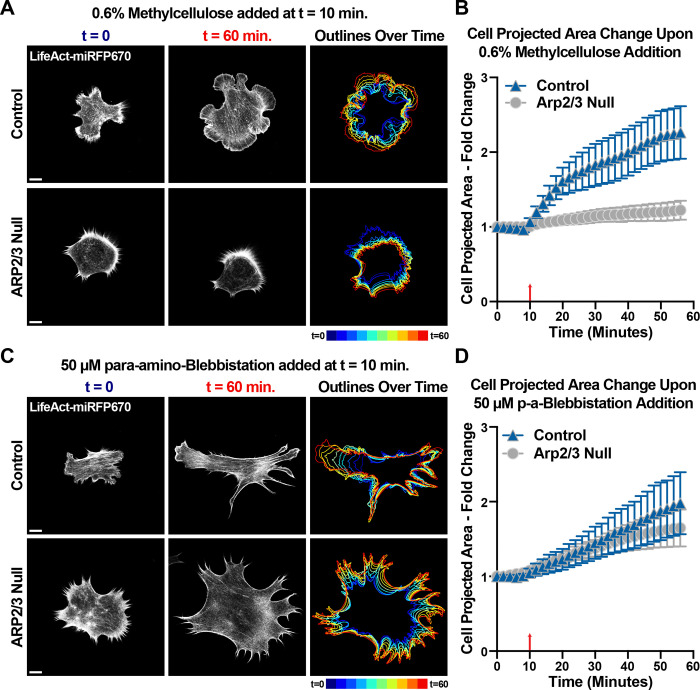
Robust spreading in response to increased extracellular viscosity requires Arp2/3-branched actin. **(A)** Frames from time lapse movies of lentiviral transduced LifeAct-miRFP670 expressed by control and 4-HT-treated *Arpc2* knockout Fibroblasts plated on glass coverslips coated with poly-L-Lysine and treated with 50 μM para-amino-Blebbistatin at t = 10 minutes. Scale bars span 10 microns. **(B)** Plot of projected cell spread area over time measured among cells shown and described in (A), with the red arrow at t = 10 minutes marking when wash-ins were performed. n = 27 for control and 28 for Arp2/3 Null cells from 2 experiments. **(C)** Frames from time lapse movies of lentiviral transduced LifeAct-miRFP670 expressed by control and 4-HT-treated *Arpc2* knockout Fibroblasts plated on glass coverslips coated with poly-L-Lysine and treated with 0.6% methylcellulose at t = 10 minutes. Scale bars span 10 microns. **(D)** Plot of projected cell spread area over time measured among cells shown and described in (A), with the red arrow at t = 10 minutes marking when wash-ins were performed. n = 33 for control and 32 for Arp2/3 Null cells from 2 experiments for each condition.

Considering the dramatic increase in branched actin density observed in cells when spreading on poly-L-Lysine in a high viscosity environment, we reasoned that this behavior might require Arp2/3 actin branching. Upon loss of Arp2/3 through Cre-mediated recombination at the *Arpc2* locus, we did not observe any spreading of the cells in response to methylcellulose treatment (Fig.6A-B). Neither WT nor Arp2/3 null cells showed any change in cell area upon treatment with methylcellulose when already spread out on fibronectin (Fig.6S1). However, loss of Arp2/3 did not prevent cell spreading on poly-L-Lysine in response to bleb treatment (Fig.6C-D), likely due to differences in spreading mechanisms. Thus, cell shape changes in response to increased hydraulic resistance to protrusion requires the Arp2/3 complex and the dense branched actin networks it generates.

### Viscosity-induced spreading on PLL-coated soft substrates generates smaller traction forces than spreading via robust Integrin-ECM engagement.

Cells spreading on fibronectin generate integrin-based adhesions that link contractile and protrusive actin networks to the ECM(Parsons, Horwitz et al. 2010). When integrin adhesions are engaged near the protrusive leading edge of cells, they can resist the retrograde flow of treadmilling actin networks, and this generates traction forces on the substrate driving cell spreading and shape changes. Upon bleb treatment, relieving cortical contractility that resists cell shape change might reduce the amount of traction forces required to drive cell spreading. However, the extent to which viscosity-induced spreading on poly-L-Lysine requires or influence cellular traction forces is not known. The scarce integrin ligand availability under such conditions would suggest that clutching engaged with the substrate is unlikely to produce enough of the force needed to facilitate cell flattening and spreading. To test this this, we employed time-lapse traction-force microscopy of MEFs plated on ~20 kPa PDMS substrates to examine how increased viscosity-induced spreading on poly-L-Lysine compared to integrin-based spreading on fibronectin coating.

**Figure 7.**
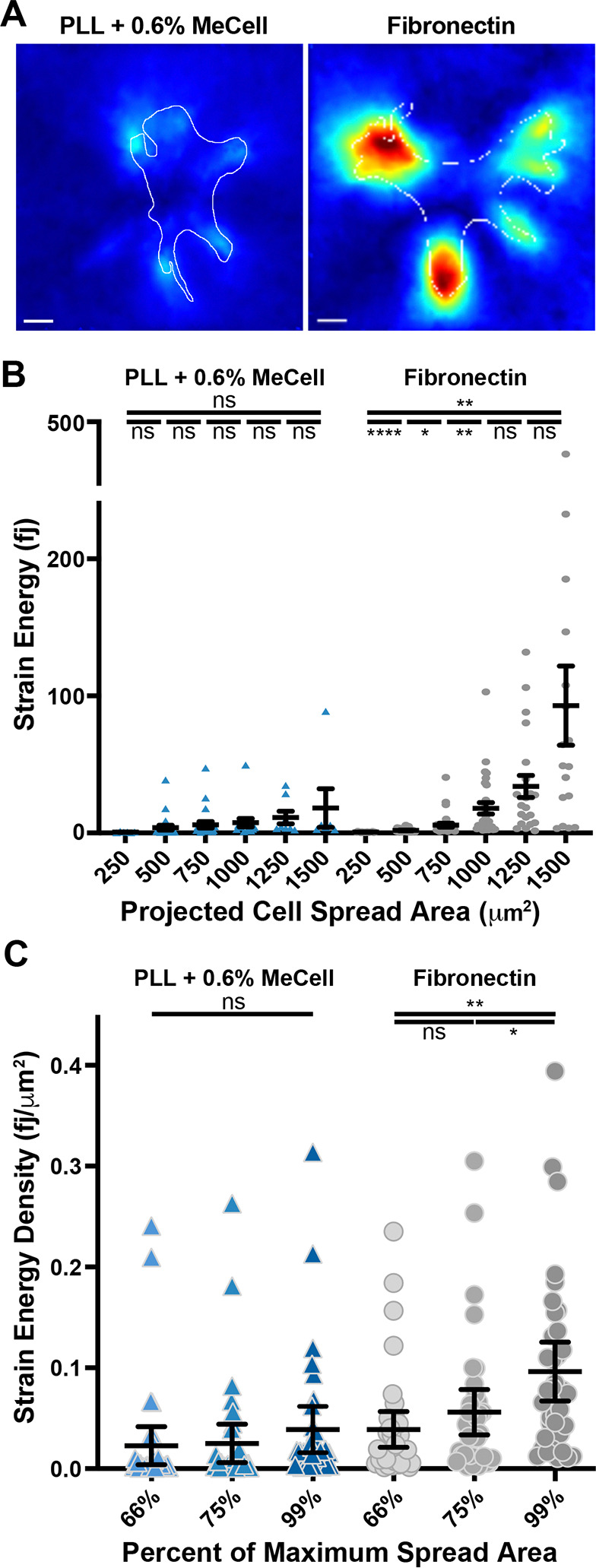
Viscosity-induced spreading on PLL-coated soft substrates generates smaller traction forces than spreading via robust Integrin-ECM engagement. **(A)** Traction maps generated from representative still images selected from time-lapse movies of fibroblasts plated on 20 kPa PDMS substrates with red fluorescent beads attached to the surface allowing for TFM measurements and coated with either poly-L-Lysine or fibronectin. Methylcellulose was added at a concentration of 0.6% to promote spreading on the poly-L-Lysine coated substrates with similar dynamics to the cells plated on fibronectin. **(B)** Plot of changes in strain energy as cells spread out under the two conditions shown and detailed in (A), with error bars representing the mean standard error of the mean. For spread areas of 250, 500, 750, 1,000, 1,250 and 1,500 square microns, n = 6, 23, 22, 15, 8 and 6 cells for PLL + 0.6% Methylcellulose and 18, 29, 32, 29, 21 and 17 cells for FN plating conditions, respectively, from 3 experiments. **(C)** Plot of the strain energy density of forces applied by the same cells measured in (B) as they reached 66%, 75%, and 99% of the maximum recorded projected spread area.

For untreated cells plated fibronectin-coated PDMS, we measured traction forces that were considerably higher on traction maps than those measured for cells plated on poly-L-Lysine coated PDMS and treated with 0.6% methylcellulose (Fig.7A). Under both conditions, cells spread out significantly over the course of an hour, but while the strain energy applied to substrate does not significantly increase during viscosity-induced spreading on poly-L-Lysine, it does when cells are spreading over dense fibronectin coating (Fig.7B). We also found significant increases in strain energy density among cells plated on fibronectin-coated PDMS when spreading from 66% to 75% final spread area as well as from 75% to 99%, and such significant increases were not similarly seen when cells were spreading on poly-L-Lysine following methylcellulose addition (Fig.7C).

### Cell protrusions generated via optogenetic Rac activation are facilitated by increased extracellular viscosity on ECM-deficient substrates coated with poly-L-Lysine.

**Figure 8.**
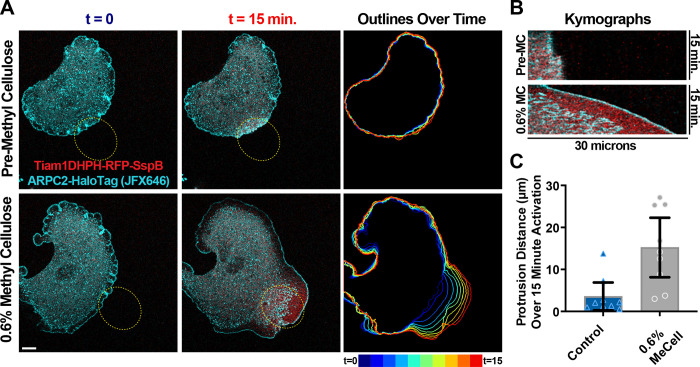
Cell protrusions generated via optogenetic Rac activation on substrates coated with poly-L-Lysine are facilitated by increased extracellular viscosity. **(A)** Images from timelapse movies of Tiam1-DH/PH-TagRFPt-SspBmicro and ARPC2-HaloTag (labeled with JF646 Halo Ligand) stably expressed by a 4HT-treated *Arpc2* knockout fibroblast via lentiviral transduction that has been plated on a glass coverslip coated with poly-L-Lysine and regularly stimulated with 405 nm light inside the region of interest (ROI) labeled with a yellow dashed oval starting at t = 0. These cells additionally express Venus-iLid-caax (not shown). The same cell is shown in all panels and was imaged during stimulation both before (top row) and after 0.6% methylcellulose addition (bottom row). Scale bar spans 10 microns. **(B)** Kymographs generated from a line (not shown) bisecting the long axis of the yellow dashed oval ROIs shown in (A). **(C)** Plot of the distances cells detailed in (A) protruded upon optogenetic activation of Rac either before or after addition of 0.6% methylcellulose. n = the same 9 cells across 3 experiments for both conditions.

In cells spreading on fibronectin, integrin engagement provokes both signaling and mechanical resistance to retrograde actin flow. In light of our data showing that increased extracellular viscosity (and resistance to protrusion) negates the need for integrin-based engagement for efficient cell spreading, we sought to test how signaling downstream of integrins relates to this alternate mechanical input. Optogenetic stimulation of Rac at the leading edge of cells plated on poly-L-Lysine leads to some increased ruffling, but as we saw before (Fig.2E), little to no forward protrusion or Arp2/3-branched actin enrichment (Fig.8A-B and Movie 15). However, following treatment with methylcellulose and the subsequent spreading response, Rac activation leads to large, robust protrusions pushing into stimulated regions (Fig.8A-B and Movie 16). Across multiple cells, Rac activation under increased extracellular viscosity conditions triggered robust protrusions compared to pre-treatment controls (Fig.8C). Together, these data show that resistance to protrusion through increased viscosity leads to dense Arp2/3-branched actin networks and subsequent protrusion, and this condition primes the leading edge to be sensitive to further protrusion induced by increased Rac activity.

## DISCUSSION

In the present study, we examined the relationship between cell mechanics and signaling that control protrusive Arp2/3-branched actin organization, striving to help fill the gap in our understanding of the hierarchy or overlap between these two actin regulatory inputs. The biochemical regulation of actin dynamics through signaling from integrin adhesions has been well characterized(Romero, Le Clainche et al. 2020), with Rho-family GTPases being common downstream effectors of signaling from clustered integrin adhesion complexes. The mechanical regulation of branched actin organization has been characterized through theoretical modeling and *in vitro* experiments, and some evidence for force feedback on Arp2/3-branched actin networks in cells has been documented(Papalazarou and Machesky 2021). Most mechanical manipulations of cells have focused on altering membrane tension or examining actin network components with inferred local membrane tension fluctuations during protrusion-retraction cycles. Here, we provide a comprehensive analysis of the control of actin branch dynamics through the monitoring of endogenous Arp2/3 complexes in several different experimental contexts and show through live cell imaging and genetic knockout that Arp2/3-branched actin is a key component in responding to and generating increased mechanical forces that drive cellular protrusions.

### Integrin signaling alone is insufficient to explain differences in Arp2/3-branched actin architecture

Examining cells plated on two different substrates, one supporting integrin engagement (Fn) and one without (PLL), revealed striking differences in branched actin organization, with more densely enriched branching seen when integrins are engaged. One prediction based on this observation might be that factors such as active Rac and other branch-promoting factors are diminished in the absence of integrin adhesions. However, we observed abundant WAVE NPF and Cortactin localization at the leading edge in cells plated on PLL, and we found that activating Rac in cells plated under similar conditions does not lead to a noticeable difference in branched actin organization at the leading edge. Considering optogenetic activation of Rac does not drive protrusion in cells plated in the absence of extracellular matrix proteins(Zimmerman, Asokan et al. 2017), the Arp2/3-branched actin networks that drive lamellipodial protrusion may require integrin signaling from nascent adhesions either beyond or in addition to activating Rac, such as PIP3 production downstream of FAK(Wang, An et al. 2024). However, the difference in protrusion morphology upon genetic loss of Arp2/3 supports the notion that there is an important structural role of actin branching in supporting broad protrusions on PLL, even in the absence of branch-promoting feedback signals from integrin adhesions. Thus, there seems to be a basal level of branching activity, either driven by or maintained by the preference for WAVE NPF to localize to regions of “saddle curvature” such as is found at the edge of flat protrusions curving outwards(Pipathsouk, Brunetti et al. 2021, Wu, Sadhu et al. 2025). These results encouraged us to turn our focus towards cell shape and mechanics providing force feedback on actin organization to explain the differences seen between Fn and PLL plating conditions.

### Physical spreading alone is sufficient to enrich Arp2/3-branched actin at the periphery

Having ruled out differences in adhesion signaling as the likely explanation for the striking differences seen in branched actin organization on Fn compared to PLL-coated substrates, we reasoned that mechanical feedback on actin networks from changes in membrane tension during spreading and clutching retrograde flow of the dendritic branched network by nascent adhesions should play a more prominent role. Examining cells one hour after plating on PLL, conditions supporting an absence of robust adhesions and dense actin branching, we observed incomplete spreading with excess membrane is stored in folds, ruffles, and filopodia. Indeed, waiting several hours more, long enough for cells to sufficiently spread on PLL without additional manipulations, we observed Arp2/3-branched actin enrichment. This reveals that integrin engagement is not strictly required for the dense actin branching in protrusions seen in cells plated on Fn but rather promotes efficient cell spreading. However, we could not completely rule out the secretion of additional ECM molecules besides fibronectin as being important for this delayed but eventual spreading seen on PLL.

To investigate the notion that ECM is more of a means to end - that the resulting cell shape and mechanics following efficient spreading on ECM has the strongest influence on leading edge actin organization - we promoted adhesion-independent cell flattening and spreading with manipulations that eliminated the need for traction forces to act against cortical contractility and the resistance to shape change it confers. Each of the treatment conditions tested, including blebbistatin to inhibit non-muscle myosin, physical compression under agarose, and methylcellulose to increase extracellular viscosity, all have their own unique caveats, but they share a similar outcome in increased branched actin enrichment in protrusions. All three of these treatments result in robust spreading of cells, which has been shown to lead to an increase in membrane tension as excess membrane in ruffles and folds is flattened out during the spreading process (Gauthier, Fardin et al. 2011). It is likely the shape cells adopt when efficiently spreading on dense ECM has a strong influence on branched actin density, whether through causing more confined, flattened protrusions, increasing membrane tension as cells increasingly spread out, or a likely contribution of both of these factors, which does not necessary rely upon on ECM engagement specifically for dramatic changes in branched actin organization.

### Branched actin force generation supports integrin-independent cell spreading

When plated on PLL, adhesions in our model cell lines are greatly reduced or absent, and traction force increases during robust spreading upon increasing extracellular viscosity when cells are plated on PLL are negligible in comparison to cells spreading with focal adhesions on dense ECM. This leads to the question then, how MEFs are generating the forces needed to spread and flatten upon viscosity-induced spreading on PLL if not applying significantly increased traction forces to the substrate. Due to the dramatic increase in branched actin density seen in our cells upon increasing extracellular viscosity, we reasoned this response we observed may be important for the concomitant cell spreading and flattening behavior. Rather than rely on pharmacological inhibitors, we were able to employ conditional *Arpc2* knock-out due to the genetic background from which the MEF cell lines utilized here were isolated (Rotty, Brighton et al. 2017) to investigate this further, and we found actin branching was indeed required for this robust response to changes in viscosity.

More dense networks with more lateral coherence are likely to encounter significantly increased resistance to retrograde flow by the viscous cytoplasmic environment, especially when flowing through a more crowded and confined flat protrusion in more spread out cells. We suggest that there may be an emphasized role for resistance to protrusive actin retrograde flow by the cytoplasmic environment specifically when the network is highly branched, which occurs in response to external stimuli that increase actin-membrane interface stress by offering mechanical resistance to protrusion. This cytoplasmic resistance to polymerizing branched actin has been characterized as an important intracellular force generator in several contexts, driving processes such as endomembrane fission(Derivery, Sousa et al. 2009, Gomez and Billadeau 2009, Marchan and Bear 2025).

### Actin-membrane interface stress as a critical regulatory input to Arp2/3-branched actin

Unique amongst our treatments, the addition of sorbitol to introduce hyper-osmotic pressure led to less cell spreading and left excess membrane folds on the top surface of the cell intact but *still* increased the density of Arp2/3-branched at the periphery. This suggests that the key factor for the enrichment of Arp2/3-branched actin involves increased stress between the barbed ends of polymerizing actin filaments at the leading edge against the cell membrane. We postulate that this actin-membrane interface stress is directly influencing branched actin density in protrusions, producing increased Arp2/3-branched actin density to produce more protrusive forces upon meeting greater resistance to protrusion. Increased stress between the plasma membrane and the barbed ends of actin filaments could limit accessibility of profilin-actin to sites of preferred monomer addition, which along with capping protein(Funk, Merino et al. 2021), shift actin polymerization away from simple barbed end elongation and towards increased branching density.

It is interesting to consider other contexts besides cell spreading and protrusion where the concept of actin-membrane interface stress would apply. Branched actin has been shown to increasingly accumulate at sites of frustrated endocytosis(Wang, Galletta et al. 2016, Yang, Colosi et al. 2022), cell-cell junctions(Del Signore, Cilla et al. 2018, Efimova and Svitkina 2018, McEvoy, Sneh et al. 2022), and sites of endomembrane fission (Marchan and Bear 2025), though much less is known about the orientation of branched actin polymerization in these contexts and where, specifically, the sites of contact are at which stress is applied on the barbed ends of the branched network. In addition, it likely that this concept has important implications throughout development and disease, though few examples have been well characterized due to the difficulty of manipulating and measuring branched actin force responsiveness *in vivo*. In one potential example, disrupting matrix metalloproteinases activity *C. elegans* embryos leads to an irregular and delayed migration of the anchor cell through the basement membrane that involves enrichment of Arp2/3-branched actin to help the cell physically force its way through (Kelley, Chi et al. 2019).

### The relationship between cell mechanics and signaling as inputs to Arp2/3-branched actin

To summarize, while both cell signaling and cell mechanics are important regulatory inputs for branched actin regulation, cell spreading, and protrusive migratory behaviors, it appears that the mechanics of actin-membrane interface stress are most influential for direct effects on branched actin architecture. Our results show Arp2/3 plays a structural role for flat, broad protrusions, even in the absence of integrins, that is supported by branch-promoting factors. In addition, Rac activation is not sufficient to increase actin branching without sufficient mechanical support or input, which suggests that biochemical signaling from integrin adhesions may be more important for protrusion persistence, coherence, or other regulatory behaviors that might support cell polarization, adhesion dynamics, and traction generation. In support of this model, we demonstrate here that the Arp2/3-dependent response to increased extracellular viscosity was sufficient to circumvent the need for ECM to drive cell protrusion upon optogenetic Rac activation. These results are likely related to previous studies done using leukocytes that showed a necessity for integrins for 2D migration on flat surfaces but not 3D migration in more confined and restrictive environments (Lämmermann, Bader et al. 2008), and we theorize that integrin-deficient leukocytes in the restricted, 3D context would likely be more responsive upon Rac or Cdc42 activation. Future studies will be needed to understand in more detail how actin-membrane interface stress or sufficient resistance to protrusion are necessary for signaling inputs such as Rac GTPase to trigger robust Arp2/3-branch generation.

## MATERIALS AND METHODS

### Cell Culture and Standard Plating Conditions

Cell lines were cultured and imaged in DMEM (4.5 g/L D-Glucose, L-Glutamate, Sodium Pyruvate, Gibco, cat. no. 11995–065) supplemented with 10% FBS (MedSupply Partners) and 1x GlutaMax (Gibco, cat. no. 35050–061) at 37°C with 5% CO_2_. Cell lines used in experiments tested negative for mycoplasma using a commercial detection kit (InvivoGen). For general imaging conditions, glass bottom dishes (Cellvis, cat. no. D35–20-1.5-N) were coated with 0.01% poly-L-Lysine solution (Millipore Sigma, cat. no. P4707) or Human fibronectin (Corning, cat. no. 356008) dissolved at 10 μg/ml in PBS for 30 minutes at room temperature before washing with PBS. MEFs were lifted from culture dishes using 0.25% Trypsin-EDTA (Gibco, cat. no. 25200056), and approximately 25,000 (Attofluor A-7816 imaging chambers) or 50,000–75,000 (Cellvis glass bottom dishes) cells were seeded for at least 30 minutes prior to imaging. To induce recombination at the *Arpc2* locus, 2 μM of 4-Hydroxytamoxifen (Millipore Sigma, cat. no. H6278) was added to cultured cells at least one week before any experiments examining Arp2/3 null phenotypes.

### Confocal Imaging

Images were captured on a Zeiss LSM800 confocal microscope fitted with a Tokai Hit stage top and Pecon large chamber incubators using a Plan-Apochromat 63X/1.4 NA Oil objective. Images were typically captured at 1024×1024 pixels using 2–8x averaging and less than 0.5% of 10 mW maximum laser power for excitation. For measurements of retrograde actin flow, GFP-β-Actin was bleached using 100% power 488 nm laser, and for optogenetic stimulations, 0.2% power of this same laser was used to stimulate the ROI between scans. Fixed cells for confocal imaging were prepared by treating with 4% PFA premixed into fresh culture media and incubating at room temperature for 10 minutes. For F-actin visualization, ﬁxed cells were washed 3x in PBS, permeabilized with 0.2% Triton-X in PBS for 5 minutes at room temperature, and then stained with Alexa Fluor 647 Phalloidin (ThermoFisher, cat. no. A2287).

### Cell Manipulations

Agar compression was performed by casting 1% agarose dissolved in culture medium in imaging chambers (Attofluor A-7816), gently sliding the agar puck over cells plated in glass-bottom dishes, and placing ~8 grams of weight on top of the agar puck during a pause in image acquisition. Bleach correction for ARPC2-mScarlet line scans at the leading edge was performed by normalizing to the background intensity regions around the nucleus. Addition of para-amino-Blebbistatin (Cayman Chemical, cat. no. 22699), Sorbitol (Sigma, cat. no. S1876), and Methylcellulose (R&D systems, cat. no. HSC001) was performed by adding 1 ml total of treatment diluted in media to the 1.5 ml of media present in glass-bottom imaging dishes.

### Barbed End Assay

The barbed ends of free actin filaments were labeled as previously described(Bryce, Clark et al. 2005), but with slight modifications. MEFs were plated into glass-bottom dishes prepared as described above and allowed to settle for 2 hours prior to labeling and fixation. Cells were washed with pre-warmed PBS and then permeabilized and labeled with 3.2 μM Alexa Fluor 488-labeled actin (Life Technologies, cat. no. A12373) in permeabilization buffer (20 mM HEPES, 138 mM KCl, 4 mM MgCl2, 3 mM EGTA, 0.2 mg/mL saponin, 1% BSA, 1 mM ATP, 3 μM phalloidin) for 30 seconds. The cells were then immediately fixed using 4% paraformaldehyde and washed in PBS prior to imaging.

### Protein Purification and Coverslip Coating

To purify RGD peptides for coating glass coverslips, sequences coding for either Hisx6-SspB-PHSRNSGSGSGSGSGRGDNP or Hisx6-SspB-GRGDS were cloned into pQE-80L vector via Gibson Assembly, and the resulting plasmid was transformed into AVB101 competent cells (Avidity, cat. no. CVB101). Cultures were grown in autoinduction media (Novagen, cat. no. 71757) supplemented with 50 μM biotin (Sigma, cat. no. B4639) overnight at 18 degrees, and pelleted bacteria were frozen, thawed, resuspended and sonicated prior to protein extraction using Ni-NTA purification spin columns (ThermoFisher, cat. no. 88229) according to manufacturer’s protocol.

For coating of glass with purified peptides, coverslips were washed with methanol and plasma cleaned for 5 minutes following drying. The coverslips when the immersed in 5% (v/v) (3-glycidyloxypropyl)trimethoxysilane (GLYMO, Sigma, cat. no. 440167) solution in MeOH for 16 h at room temperature. The following day, coverslips when then washed with isopropanol and heated at 105 degrees Celsius for one hour. Once cooled, the coverslips were coated with 1 mg/ml NeutrAvidin (ThermoFisher, cat. no. 31000) diluted in PBS overnight at room temperature in the dark. The following day, coverslips are washed with PBS and coated for 2 hours with 2 mg/ml of purified protein.

### Scanning Electron Microscopy

For scanning electron microscopy (SEM) imaging, cells where plated on round 12 mm diameter coverslips and fixed with 2.5% glutaraldehyde/0.15M sodium phosphate buffer (pH 7.4) for one hour at room temperature. Cells were post-fixed for 15 min with a buffered 1% osmium tetroxide (washed 3x in water) following by 2% tannic acid in water for 15min (washed 3x in water) and then 15m in aqueous 1% osmium tetroxide. The samples were washed in a final 3 steps exchanges of deionized water for 10 min each. Samples were then dehydrated in a gradient ethanol series and critical point dried using CO2 as transitional solvent (Samdri-795 critical point dryer, Tousimis Research Cop., Rockville, MD). Coverslips were then mounted and sputtered coated with 5 nm gold-palladium alloy (60Au:40Pd) using a Cressington 208HR Sputter Coater (Ted Pella Inc.). Images were acquired on a Zeiss Supra 25 FESEM (Carl Zeiss SMT Inc.) operating at 5 kV using a 6 mm working distance and 20 μm aperture.

### Traction Force Microscopy

Traction force microscopy was performed similarly to as previously detailed (Teo, Lim et al. 2020). In brief, a solution of compliant PDMS was prepared by mixing rigid Sylgard 184 (Dow, cat. no. 1317318) and compliant Sylgard 527 (Dow, cat. no. 1696742). Sylgard 184 was prepared at 10:1 (Part A to Part B by weight) and mixed thoroughly before degassing for 15 minutes in a tabletop vacuum chamber. While degassing, a Sylgard 527 solution was prepared at 1:1 (Part A to Part B by weight), mixed thoroughly and degassed for 15 minutes in a tabletop vacuum chamber. Once both solutions are degassed, they were mixed in a 20:1 ratio (Sylgard 527 to Sylgard 184) by weight and mixed thoroughly before degassing for 15 minutes in a tabletop vacuum chamber. When cured, this mixture produces substrates that are approximately 20 kPa as determined by indentation analysis(Hockenberry, Ulmer et al. 2025). Once degassed, approximately 25 μL of the compliant PDMS mixture was immediately added to a plasma cleaned #1.5 25 mm coverslip and placed in a spin coater and ran for 30 seconds at 3000 RPM to produce an approximately 50-micron thick flat surface. The substrate was then placed in an empty pipette tip box and put into a 60 C oven for at least 24 hours to fully cure the PDMS.

To add fluorescent beads to the substrates, we washed the cured PDMS with 100% EtOH. We then treated the surface with 2 mL of a 10% by volume APTES (Thermo, cat. no. 430941000) in 100% EtOH for 30 minutes on a shaker table. The APTES solution was removed from the substrates, and then they were washed 2x with 100% EtOH before 2 mL of a 1/1000 solution of carboxylate polystyrene fluorescent beads (Thermo, cat. no. F8801) and 1 mg/mL EDC (ThermoFisher, cat. no. E7750) in diH20 was added to the substrates, which were then allowed to rock on a shaker table for one hour. The bead and EDC solution was then removed, and the surface was washed with diH20. Substrates were either used immediately or stored for up to one week.

To add fibronectin or poly-L-Lysine and sterilize the substrates, we placed the substrates upside down into a fresh, sterile plastic 6-well dish containing 1 mL of 10 μg/mL of fibronectin (Gibco 33010018) or 0.1 mg/mL of Poly-L-Lysine (Millipore Sigma cat. no. P4707) inside of a tissue culture hood. The plastic 6-well dish was then irradiated with UV light for 30 minutes to sterilize the substrates while the proteins coated the surface. Once irradiated, the substrates were washed with sterile PBS 3x times.

Substrates were placed into a 35 mm metal imaging dish and 1 mL of culture media containing approximately 5,000 JR20 Fibroblasts stably expressing eGFP-Paxillin were added before a sterile plastic lid from a 35 mm plastic cell culture dish was used to cover the dish. Cells were imaged for 1 hour, with snapshots being taken every thirty seconds. Cells plated on PLL were allowed to adhere for one hour before imaging for one hour after selecting positions. After timelapse imaging, 100 μL of 1% SDS in DMEM was added to the substrate to remove the cells and then a reference state was taken of the cell free bead field.

### Traction force analysis

Traction force analysis was performed using μ-inferforce, a MATLAB TFM package (Han, Oak et al. 2015), as previous described (King, Butler et al. 2022). Briefly, images were drift corrected through Efficient Subpixel Registration before displacement fields were calculated grid points and PIV suite. The template size was set to 11 pixels and maximum displacement to 20 pixels. Displacement fields were corrected through filtering vector field outliers with a normalized displacement residual of 2. Force fields were constructed using FTTC, a young’s modulus value of 20, and a gel thickness of 50. A constant regularization parameter of 1e-5 was used for all images.

Traction maps were analyzed by masking the image by a 5-micron dilated cell boundary determined from the GFP paxillin channel. Masks of the cell were obtained by manually segmenting the cell boundary from the GFP paxillin channel in FIJI. The values for the traction magnitude and strain energy were computed as described in (Butler, Tolić-Nørrelykke et al. 2002).

### Image quantification and presentation

The Fiji distribution of ImageJ was used to measure areas, rates, and intensities from imaging datasets. Mean intensities along cell edges were measured with a freehand line roughly 1 micron wide at and within the cell edge of the labeled regions of the largest cell protrusion. Line scans were drawn perpendicular from the edge near the middle of the largest protrusion inward, avoiding regions with obvious ruffles and folds to try to capture regions of flat, even lamellipodia. Retrograde flow rates were measured from slopes of recovering signal in kymographs generated following bleaching of GFP-β-actin expressed via lentiviral transduction at the edge of lamellipodial protrusions. The density of GFP-Paxillin foci was determined by manually counting foci that appeared roughly 100–300 nm in diameter within ~2 microns along the largest protrusion edge. The “detect particles” tool was used following manual thresholding for cell segmentation, and mean cell intensities of segmented cells and subsequent measures using macros to reduce the ROI size in one pixel steps was used to calculate whole-cell FWHM and Max/Mean intensities. The kymograph tool was used for the generation of kymographs.

Raw images were copied from Fiji, and the Despeckle, Gaussian Blur (Radius: 0.5 pixels), and Unsharp Mask filters were applied in Photoshop (Adobe) before assembling figures using Illustrator (Adobe). Brightness and contrast were also adjusted in Photoshop and similarly applied to both control and experiment images across datasets. Prism (GraphPad) was used to plot data with 95% Confidence Interval (unless otherwise noted in the figure legend) and to perform statistical analyses. Significance notations in figures represent p ≤ 0.05 as *, p ≤ 0.01 as **, p ≤ 0.001 as ***, and p ≤ 0.0001 as ****.

## MATERIALS AVAILABILITY

All raw data, measurements and quantifications spreadsheets, scripts, and novel cell lines and plasmids are available upon reasonable request.

## Supplementary Material

Supplement 1

Supplement 2

Supplement 3

Supplement 4

Supplement 5

Supplement 6

Supplement 7

Supplement 8

Supplement 9

Supplement 10

Supplement 11

Supplement 12

Supplement 13

Supplement 14

Supplement 15

Supplement 16

Supplement 17
