# Supplementary material for: Actin-membrane interface stress regulates Arp2/3-branched actin density during lamellipodial protrusion
Source: bioRxiv. 2026 Mar 16:2026.03.06.710140. Preprint. [Version 2] doi: 10.64898/2026.03.06.710140 (PMC13015326; doi:10.64898/2026.03.06.710140)
Supplement: Supplement 17 [file NIHPP2026.03.06.710140v2-supplement-17.pdf]

bioRxiv preprint doi: https://doi.org/10.64898/2026.03.06.710140; this version posted March 16, 2026. The copyright holder for this preprint (which was not certified by peer review) is the author/funder, who has granted bioRxiv a license to display the preprint in perpetuity. It is made available under aCC-BY 4.0 International license.

Figure 1 Supplement 1

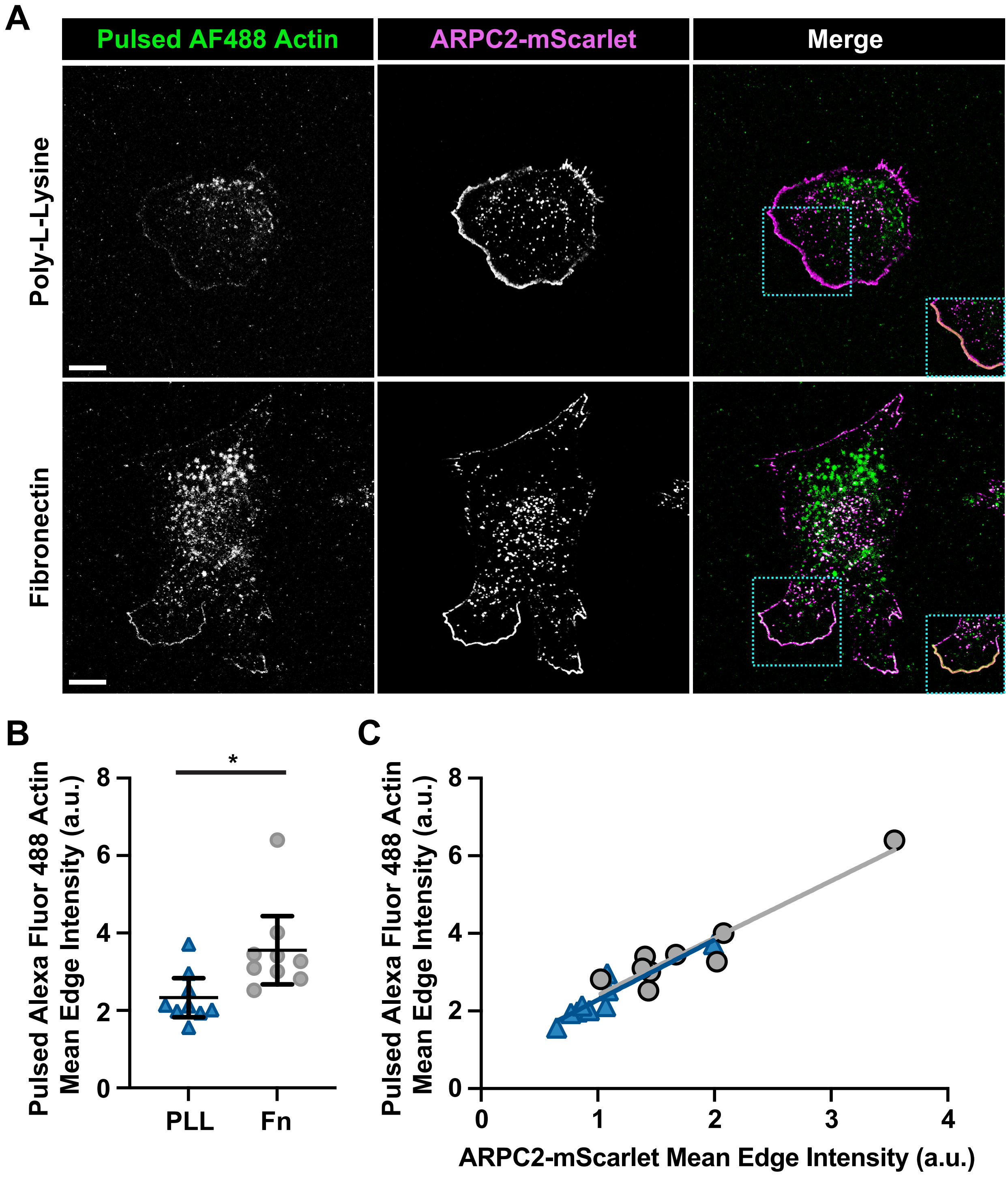

bioRxiv preprint doi: https://doi.org/10.64898/2026.03.06.710140; this version posted March 16, 2026. The copyright holder for this preprint (which was not certified by peer review) is the author/funder, who has granted bioRxiv a license to display the preprint in perpetuity. It is made available under aCC-BY 4.0 International license.

Figure 1 Supplement 1. Barbed end density increases with ARPC2-mScarlet enrichment in Fibroblasts on surfaces coated with dense fibronectin ECM. (A) Images of Fibroblasts endogenously expressing ARPC2-mScarlet after simultaneously pulsing labeled Alexa Fluor 488 Actin and unlabeled phalloidin under conditions that make the cells slightly permeable to labeled the barbed ends of stabilized actin filaments. Scale bar spans 10 microns. **(B)** Plot of mean intensity of Alexa Fluor 488 Actin in cells shown and described in (A). n = 9 cells for each condition. **(C)** Plot of values for mean edge intensity of pulsed labeled Alexa Fluor 488 Actin shown in (B) against the similarly measured ARPC2-mScarlet in the same cell, with linear regression lines of similar slopes demonstrating a similar relationship between barbed end and ARPC2-mScarlet densities in cells plated on poly-L-Lysine (blue dots) when compared to fibronectin (grey dots).

bioRxiv preprint doi: https://doi.org/10.64898/2026.03.06.710140; this version posted March 16, 2026. The copyright holder for this preprint (which was not certified by peer review) is the author/funder, who has granted bioRxiv a license to display the preprint in perpetuity. It is made available under aCC-BY 4.0 International license.

Figure 1 Supplement 2

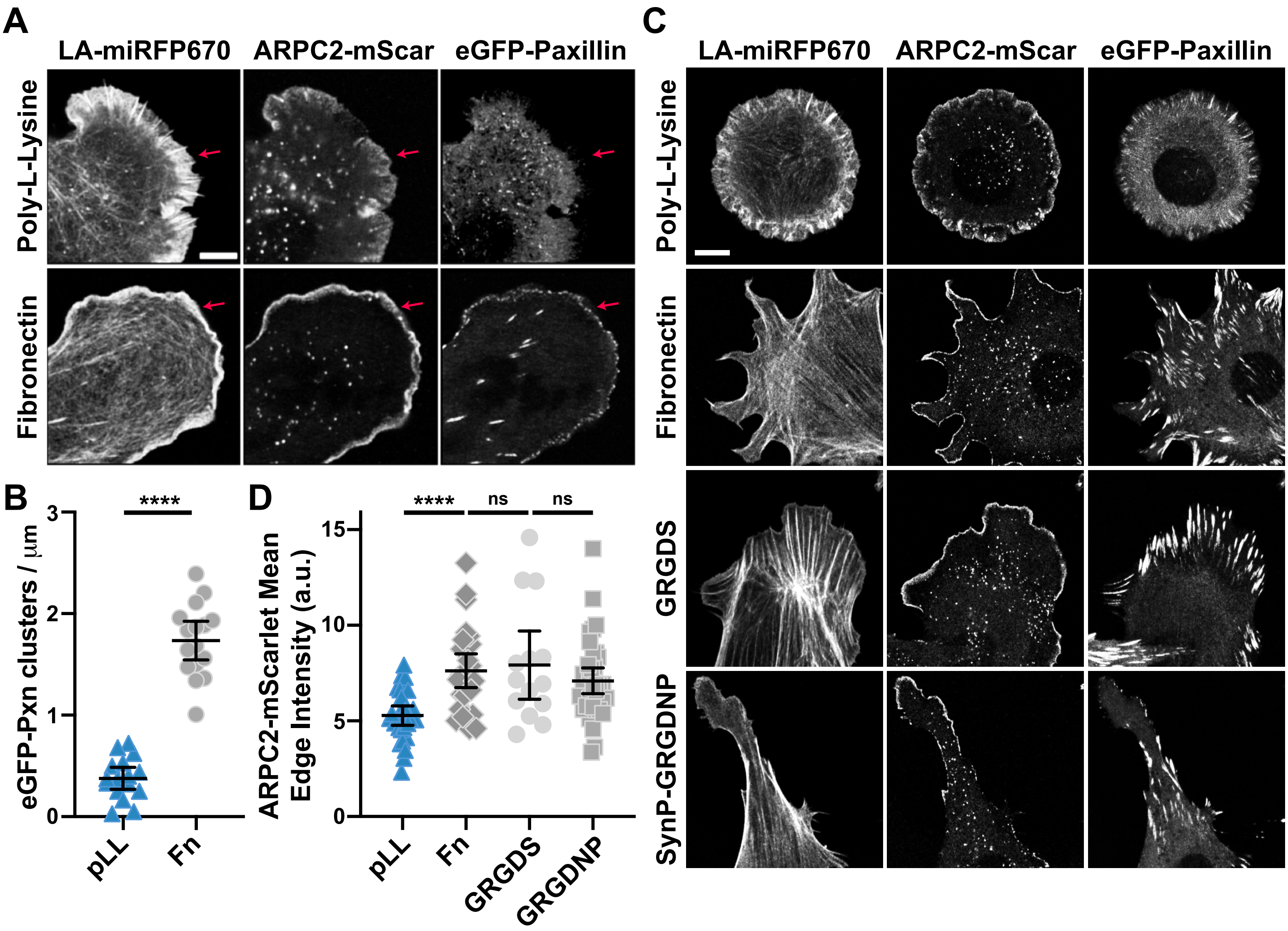

bioRxiv preprint doi: https://doi.org/10.64898/2026.03.06.710140; this version posted March 16, 2026. The copyright holder for this preprint (which was not certified by peer review) is the author/funder, who has granted bioRxiv a license to display the preprint in perpetuity. It is made available under aCC-BY 4.0 International license.

Figure 1 Supplement 2. Branched actin enrichment in fibroblasts plated on fibronectin depends on Integrin engagement. (A) Images of Endogenous ARPC2-mScarlet and lentiviral transduced LifeAct-miRFP670 and GFP-Paxillin expressed by Fibroblasts plated on surfaces that have been coated with either poly-L-Lysine or fibronectin. Red arrows mark the edge of the largest cell protrusion where the presence or absence of small paxillin clusters can be seen. Scale bar spans 5 microns. **(B)** Plot of density of GFP-Paxillin clusters around the edge of the largest cell protrusion under the conditions shown in (A). n = 16 cells from 3 experiments. Note that the data from cells on poly-L-Lysine coated glass in panels (A & B) were collected at the same time as other experimental conditions for a separate study, and the images and values for the fibronectin condition here have been published as the 10 ug/ml control condition previously . **(C)** Images of Endogenous ARPC2-mScarlet and lentiviral transduced LifeAct-miRFP670 and GFP-Paxillin expressed by Fibroblasts plated on surfaces that have been coated with covalently-linked poly-L-Lysine, fibronectin, purified GRGDS peptides, or purified PHSRNGRGDNP. Scale bar spans 5 microns. **(D)** Plot of mean ARPC2-mScarlet intensities at and within roughly 1 micron of the cell edge along the largest protrusion in fibroblasts plated as described and shown in (C). n = 29, 27, 14 and 38 for pLL, Fn, GRGDS, and GRGDNP, respectively.

bioRxiv preprint doi: https://doi.org/10.64898/2026.03.06.710140; this version posted March 16, 2026. The copyright holder for this preprint (which was not certified by peer review) is the author/funder, who has granted bioRxiv a license to display the preprint in perpetuity. It is made available under aCC-BY 4.0 International license.

Figure 3 Supplement 1

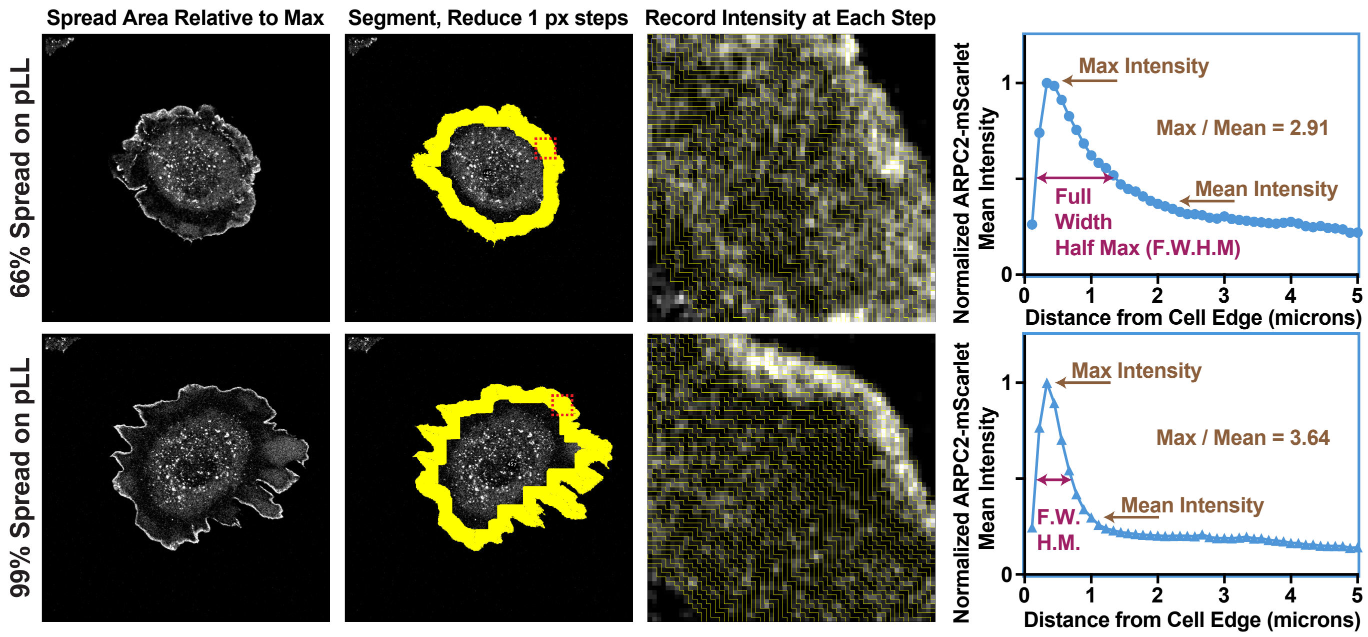

bioRxiv preprint doi: https://doi.org/10.64898/2026.03.06.710140; this version posted March 16, 2026. The copyright holder for this preprint (which was not certified by peer review) is the author/funder, who has granted bioRxiv a license to display the preprint in perpetuity. It is made available under aCC-BY 4.0 International license.

Figure 3 Supplement 1. Segmentation and quantification of Arp2/3 distribution near the cell periphery during cell spreading. Representative images of ARPC2-mScarlet endogenously expressed by a fibroblast plated on a glass coverslip coated with poly-L-Lysine as it reached a projected cell spread area of 66% (top) and 99% (bottom) of the recorded maximum spread area. Yellow lines represent segmentation of the cell around the outer edge and 5 microns into the cell from the edge in 1-pixel steps, which was used to calculate the F.W.H.M. and maximum/mean intensities as measures of branched actin density and distribution around the cell periphery during spreading.

bioRxiv preprint doi: https://doi.org/10.64898/2026.03.06.710140; this version posted March 16, 2026. The copyright holder for this preprint (which was not certified by peer review) is the author/funder, who has granted bioRxiv a license to display the preprint in perpetuity. It is made available under aCC-BY 4.0 International license.

Figure 3 Supplement 2

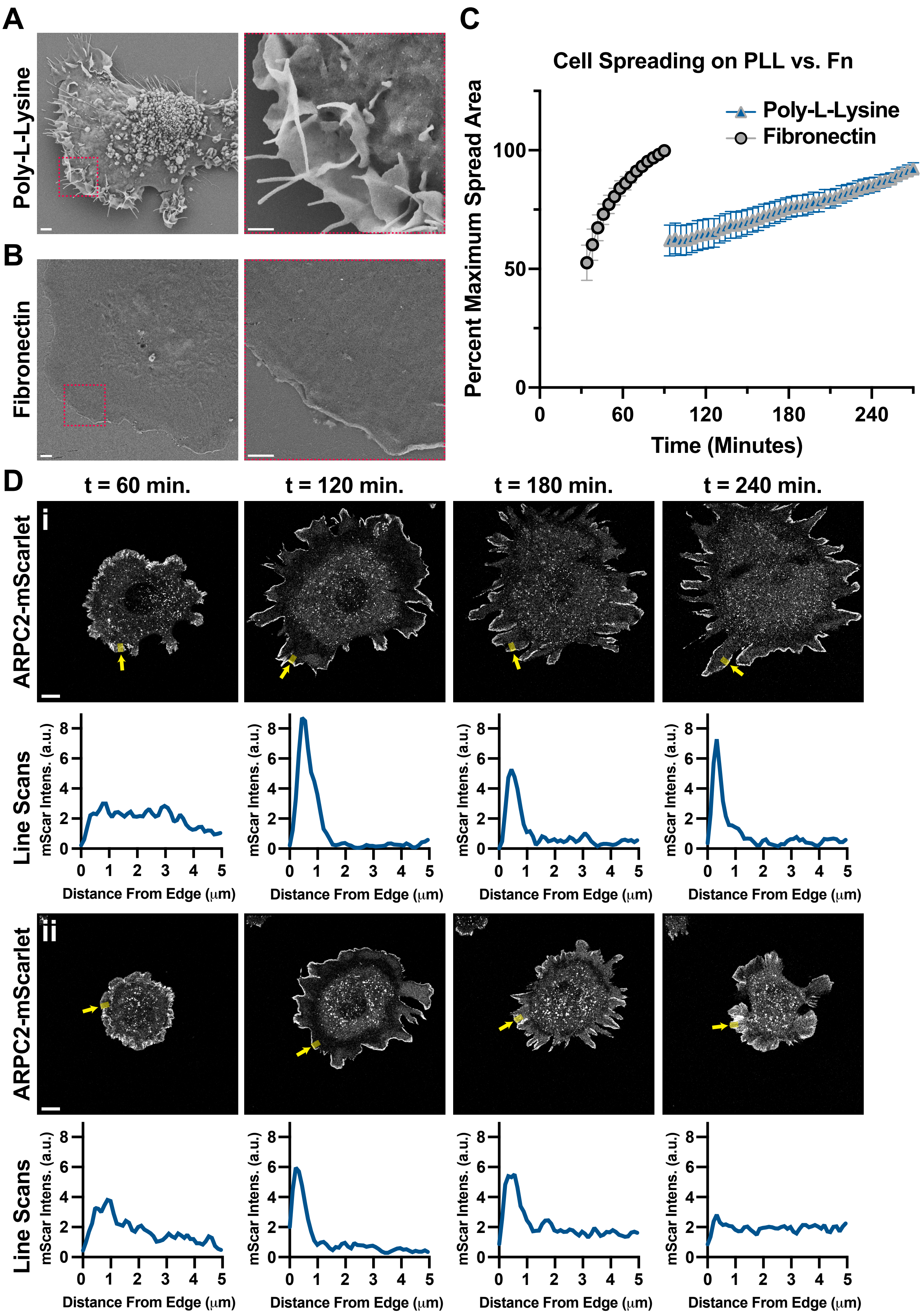

bioRxiv preprint doi: https://doi.org/10.64898/2026.03.06.710140; this version posted March 16, 2026. The copyright holder for this preprint (which was not certified by peer review) is the author/funder, who has granted bioRxiv a license to display the preprint in perpetuity. It is made available under aCC-BY 4.0 International license.

Figure 3 Supplement 2. fibronectin promotes efficient spreading and flattening of Fibroblasts. (A-B) Scanning electron microscopy (SEM) images of Fibroblasts plated on glass coverslips coated with poly-L-Lysine or fibronectin. Right panel is an enlarged view of the boxed region in the panel to the left. **(C)** Plot of projected cell spread area in fibroblasts plated on either fibronectin poly-L-Lysine over time. **(D)** Sample images and accompanying line scans measured from regions marked with yellow-shaded regions and arrows for cells plated on poly-L-Lysine. Note the maintained increase in spread area and Arp2/3-mScarlet density show in the panels for the top section **i** and both the increase and decrease of Arp2/3-density that is seen upon the increase and decrease of cell spread area shown in the panels for the bottom section **ii**.

bioRxiv preprint doi: https://doi.org/10.64898/2026.03.06.710140; this version posted March 16, 2026. The copyright holder for this preprint (which was not certified by peer review) is the author/funder, who has granted bioRxiv a license to display the preprint in perpetuity. It is made available under aCC-BY 4.0 International license.

Figure 4 Supplement 1

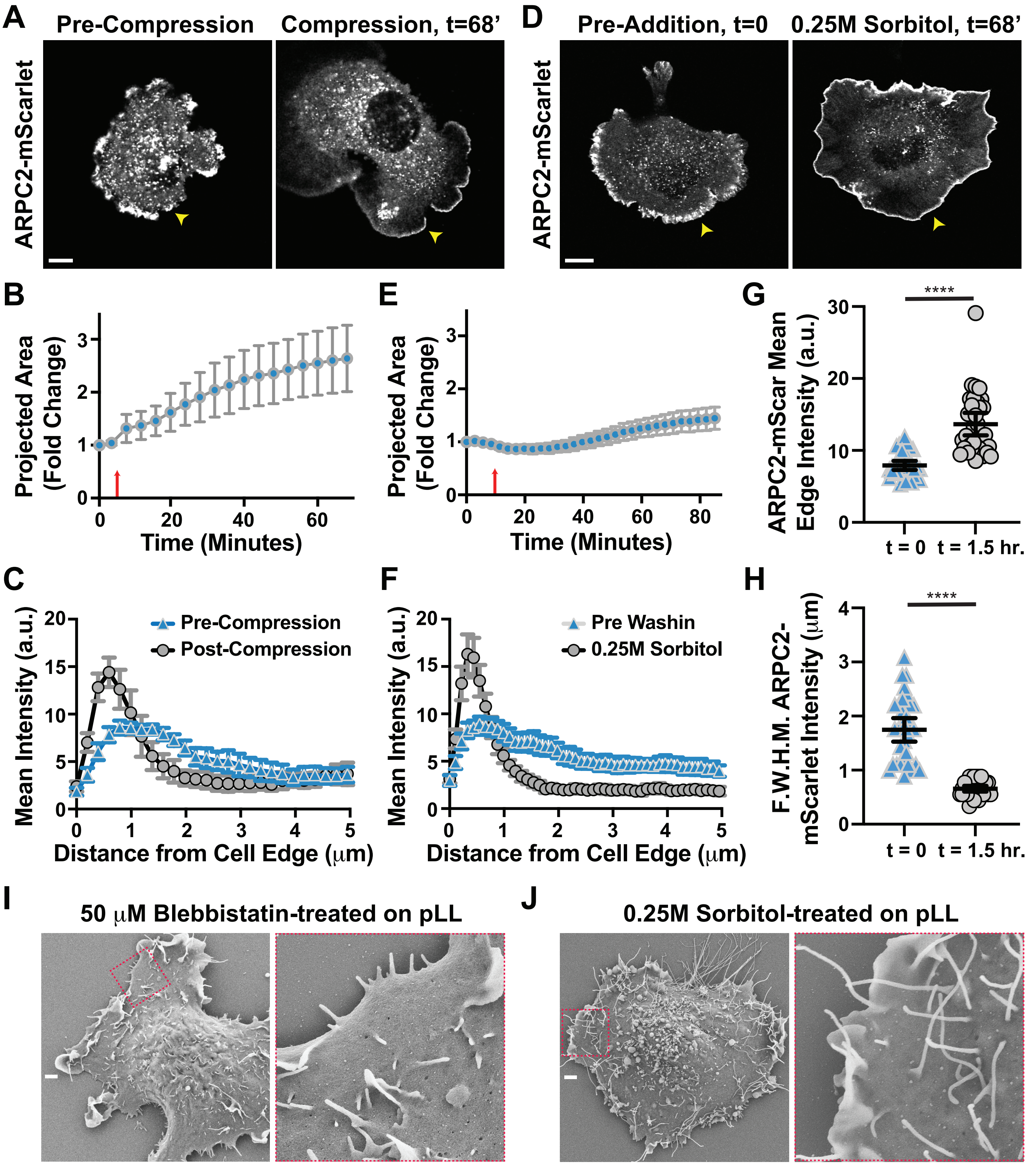

bioRxiv preprint doi: https://doi.org/10.64898/2026.03.06.710140; this version posted March 16, 2026. The copyright holder for this preprint (which was not certified by peer review) is the author/funder, who has granted bioRxiv a license to display the preprint in perpetuity. It is made available under aCC-BY 4.0 International license.

Figure 4 Supplement 1. Physically flattening cells or manipulating membrane tension with osmotic pressure leads to enriched protrusive branched actin. (A) Frames from a time lapse movie of endogenously-labeled ARPC2-mScarlet expressed by Fibroblasts plated on glass coverslips coated with poly-L-Lysine and compressed under weighted agarose pucks at t = 5 minutes to physically force cell flattening and spreading. Scale bar spans 10 microns. **(B)** Plot of the fold change in projected cell spread area after physically compressing cells at t = 10 minutes (red arrow) as shown in (A). n = 25 cells from 2 experiments. **(C)** Plot of ARPC2-mScarlet intensities along line scans starting near the middle of the largest cellular protrusion, aligned perpendicular to the cell edge, and directed inwards while avoiding regions of obvious ruffles and folds in cells plated as detailed in (A-B) following bleach correction pre- (t = 0) and post-compression (t = 60 minutes). **(D)** Frames from a time lapse movie of endogenously-labeled ARPC2-mScarlet expressed by Fibroblasts plated on glass coverslips coated with poly-L-Lysine and treated with Sorbitol at a final concentration of 0.25M at t = 10 minutes. Scale bar spans 10 microns. **(E)** Plot of the fold change in projected cell spread area after treating with 0.25M Sorbitol at t = 10 minutes (red arrow) as shown in (D). n = 32 cells from 2 experiments. **(F)** Plot of ARPC2-mScarlet intensities along line scans starting near the middle of the largest cellular protrusion, aligned perpendicular to the cell edge, and directed inwards while avoiding regions of obvious ruffles and folds in cells plated as detailed in (D-E) before (t = 0) and after (t = 90 minutes) Sorbitol addition. **(G)** Plot of mean ARPC2-mScarlet intensity around and within ~1 micron of the edge of the largest cell protrusion in cells shown and detailed in (D-F) before (t = 0) and after (t = 90 minutes) Sorbitol addition. **(H)** Plot of Full Width Half Max values calculated from the line scans shown in (F). **(I)** Scanning electron microscopy (SEM) images of Fibroblasts plated on glass coverslip coated with poly-L-Lysine and treated with 50 μM para-amino-Blebbistatin. Right panel is an enlarged view of the boxed region in the panel to the left. **(J)** Scanning electron microscopy (SEM) images of Fibroblasts plated on glass coverslip coated with poly-L-Lysine and treated with 0.25M Sorbitol. Right panel is an enlarged view of the boxed region in the panel to the left.

bioRxiv preprint doi: https://doi.org/10.64898/2026.03.06.710140; this version posted March 16, 2026. The copyright holder for this preprint (which was not certified by peer review) is the author/funder, who has granted bioRxiv a license to display the preprint in perpetuity. It is made available under aCC-BY 4.0 International license.

Figure 6 Supplement 1

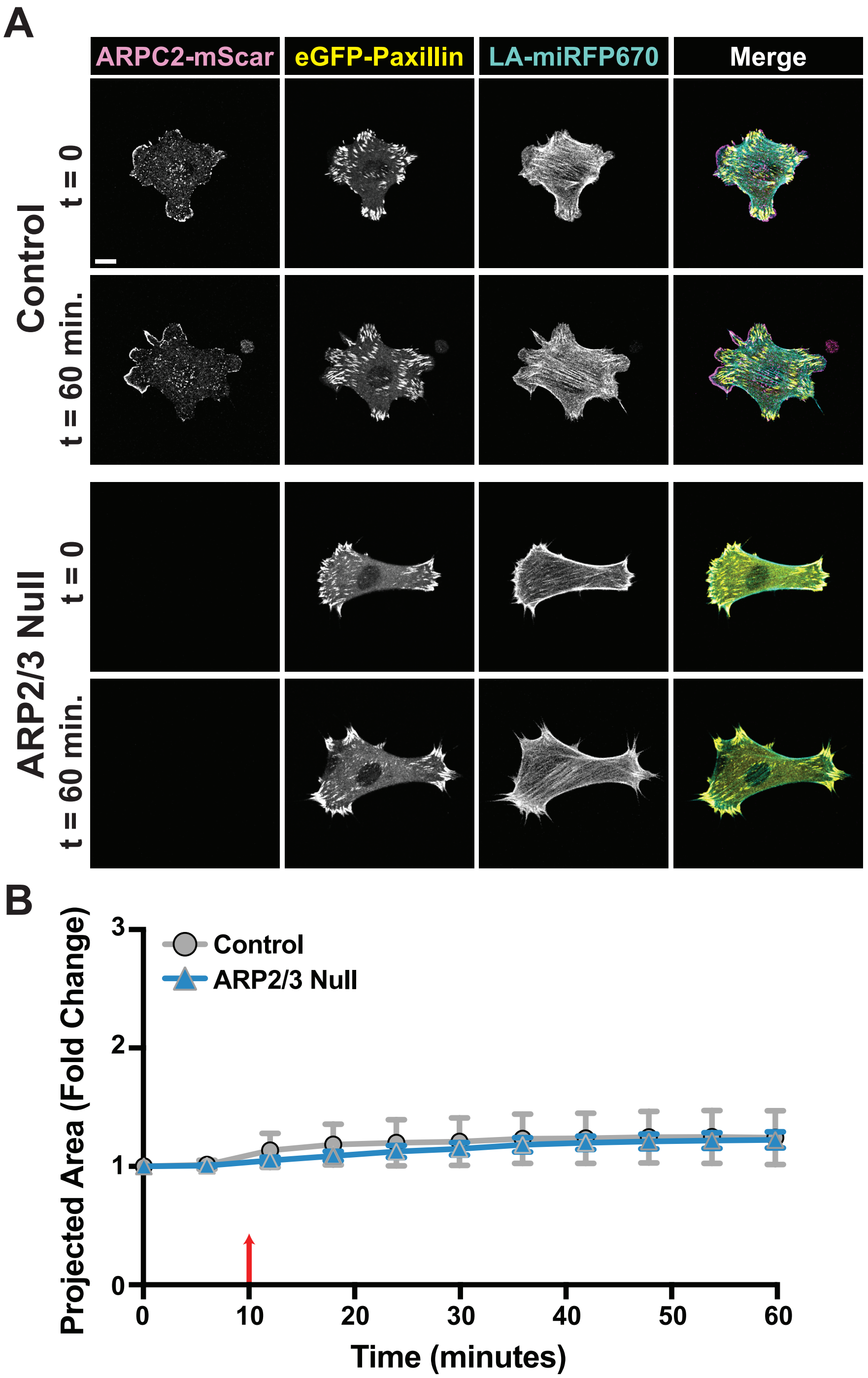

bioRxiv preprint doi: https://doi.org/10.64898/2026.03.06.710140; this version posted March 16, 2026. The copyright holder for this preprint (which was not certified by peer review) is the author/funder, who has granted bioRxiv a license to display the preprint in perpetuity. It is made available under aCC-BY 4.0 International license.

Figure 6 Supplement 1. Neither control nor Arp2/3 null Fibroblasts plated on dense fibronectin ECM exhibit robust cells spreading in response to increased extracellular viscosity. (A) Frames from time lapse movies of endogenous ARPC2-mScarlet expressed by control cells and lentiviral transduced GFP-Paxillin and LifeAct-miRFP670 expressed by both control and 4-HT-treated *Arpc2* knockout Fibroblasts plated on glass coverslips coated with fibronectin and treated with 0.6% methylcellulose at t = 10 minutes. Scale bars span 10 microns. **(B)** Plot of projected cell spread area over time measured among cells shown and described in (A), with the red arrow at t = 10 minutes marking when wash-ins were performed. n = 27 for control and 15 for Arp2/3 Null cells from 2 experiments.

SUPPLEMENTAL MOVIE LEGENDS

Supplemental Movie 1 for Figure 1A.

Representative short-term timelapse movie of endogenously-labeled Arpc2-mScarlet expressed by fibroblasts plated on glass coated with poly-L-Lysine. Scale bar spans 10 microns.

Supplemental Movie 2 for Figure 1A.

Representative short-term timelapse movie of endogenously-labeled Arpc2-mScarlet expressed by fibroblasts plated on glass coated with fibronectin. Scale bar spans 10 microns.

Supplemental Movie 3 for Figure 2A.

Representative timelapse movie of fibroblasts plated on glass coated with poly-L-Lysine while stably expressing mEmerald-WAVE1 shown both alone (left) and merged with endogenously-labeled Arpc2-mScarlet (right). Scale bar spans 10 microns.

Supplemental Movie 4 for Figure 2A.

Representative timelapse movie of fibroblasts plated on glass coated with fibronectin while stably expressing mEmerald-WAVE1 shown both alone (left) and merged with endogenously-labeled Arpc2-mScarlet (right). Scale bar spans 10 microns.

Supplemental Movie 5 for Figure 2E.

Representative timelapse movie of Tiam1-DH/PH-TagRFPt-SspBmicro (left) and Arpc2-HaloTag visualized with JF646 Halo Ligand (right) stably expressed by 4HT-treated *Arpc2* knockout fibroblasts via lentiviral transduction that have been plated on a glass coverslip coated with poly-L-Lysine. The yellow ROI shown on the first frame marks where stimulation with 405

bioRxiv preprint doi: https://doi.org/10.64898/2026.03.06.710140; this version posted March 16, 2026. The copyright holder for this preprint (which was not certified by peer review) is the author/funder, who has granted bioRxiv a license to display the preprint in perpetuity. It is made available under aCC-BY 4.0 International license.

Supplemental Movie 6 for Figure 2E.

Representative timelapse movie of Tiam1-DH/PH-TagRFPt-SspBmicro (left) and Arpc2-HaloTag visualized with JF646 Halo Ligand (right) stably expressed by 4HT-treated *Arpc2* knockout fibroblasts via lentiviral transduction that have been plated on a glass coverslip coated with fibronectin. The yellow ROI shown on the first frame marks where stimulation with 405 nm light occurred between each frame, beginning between frames taken at t = 00:13 and 00:17. See Figure 2E for scale.

Supplemental Movie 7 for Figure 3A and Supplemental Figure 2Dii.

Long-term timelapse movie of endogenously-labeled Arpc2-mScarlet expressed by a fibroblast plated on glass coated with poly-L-Lysine. Scale bar spans 10 microns.

Supplemental Movie 8 for Figure 3A.

Long-term timelapse movie of endogenously-labeled Arpc2-mScarlet expressed by a fibroblast plated on glass coated with fibronectin. Scale bar spans 10 microns.

Supplemental Movie 9 for Figure 4A.

Representative timelapse movie of fibroblasts plated on glass coated with poly-L-Lysine expressing endogenously-labeled Arpc2-mScarlet-SspB merged with stably expressed (via lentiviral transduction) GFP-Pxn that were treated with an equal volume of DMSO used for para-amino-Blebbistatin treatments, used as a control for such treatments, between frames 1 and 2. Scale bar spans 10 microns.

Supplemental Movie 10 for Figure 4A.

Representative timelapse movie of fibroblasts plated on glass coated with poly-L-Lysine expressing endogenously-labeled Arpc2-mScarlet-SspB merged with stably expressed (via lentiviral transduction) GFP-Pxn that were treated with 50 μM para-amino-Blebbistatin at t = 10:00. Scale bar spans 10 microns.

Supplemental Movie 11 for Figure 4 Supplement 1A.

Representative timelapse movie of fibroblasts plated on glass coated with poly-L-Lysine expressing endogenously-labeled Arpc2-mScarlet-SspB that were compressed under weighted agarose pucks applied at t = 5:00. Scale bar spans 10 microns.

Supplemental Movie 12 for Figure 4 Supplement 1D.

Representative timelapse movie of fibroblasts plated on glass coated with poly-L-Lysine expressing endogenously-labeled Arpc2-mScarlet-SspB merged with stably expressed (via lentiviral transduction) GFP-Pxn that were treated with 0.25M Sorbitol at t = 10:00. Scale bar spans 10 microns.

Supplemental Movie 13 for Figure 5A.

bioRxiv preprint doi: https://doi.org/10.64898/2026.03.06.710140; this version posted March 16, 2026. The copyright holder for this preprint (which was not certified by peer review) is the author/funder, who has granted bioRxiv a license to display the preprint in perpetuity. It is made available under aCC-BY 4.0 International license.

Representative timelapse movie of fibroblasts plated on glass coated with poly-L-Lysine expressing endogenously-labeled Arpc2-mScarlet-SspB merged with stably expressed (via lentiviral transduction) GFP-Pxn that were left unperturbed as controls for methylcellulose treatments. Scale bar spans 10 microns.

Supplemental Movie 14 for Figure 5A.

Representative timelapse movie of fibroblasts plated on glass coated with poly-L-Lysine expressing endogenously-labeled Arpc2-mScarlet-SspB merged with stably expressed (via lentiviral transduction) GFP-Pxn that were treated with a final concentration of 0.6% methylcellulose to increase extracellular viscosity at t = 10:00. Scale bar spans 10 microns.

Supplemental Movie 15 for Figure 8A.

Representative timelapse movie of Tiam1-DH/PH-TagRFPt-SspBmicro (red) merged with Arpc2-HaloTag visualized with JF646 Halo Ligand (cyan) stably expressed by 4HT-treated *Arpc2* knockout fibroblasts via lentiviral transduction that have been plated on a glass coverslip coated with poly-L-Lysine and stimulated in standard culture media. The yellow ROI shown on the first frame marks where stimulation with 405 nm light occurred between each frame, beginning between frames taken at t = 00:24 and 00:30. Scale bar spans 10 microns.

Supplemental Movie 16 for Figure 8A.

Representative timelapse movie of Tiam1-DH/PH-TagRFPt-SspBmicro (red) merged with Arpc2-HaloTag visualized with JF646 Halo Ligand (cyan) stably expressed by 4HT-treated *Arpc2* knockout fibroblasts via lentiviral transduction that have been plated on a glass coverslip coated with poly-L-Lysine and stimulated in media containing 0.6% methylcellulose (Note that this is the same cell shown in Movie 15). The yellow ROI shown on the first frame marks where stimulation with 405 nm light occurred between each frame, beginning between frames taken at t = 00:24 and 00:30. Scale bar spans 10 microns.
